# Structural Snapshots of π‐Arene Bonding in a Gold Germylene Cation

**DOI:** 10.1002/chem.202004566

**Published:** 2020-11-09

**Authors:** Sonia Bajo, María M. Alcaide, Joaquín López‐Serrano, Jesús Campos

**Affiliations:** ^1^ Instituto de Investigaciones Químicas (IIQ) Departamento de Química Inorgánica, and Centro de Innovación en Química Avanzada (ORFEO-CINQA) Consejo Superior de Investigaciones Científicas (CSIC), and University of Sevilla Avenida Américo Vespucio 49 41092 Sevilla Spain

**Keywords:** germylenium, gold, group 14 elements, structural snapshot, π-bonding

## Abstract

Heavier group 14 element cations exhibit a remarkable reactivity that has typically hampered their isolation. For the few available examples, the role of π‐arene interactions is crucial to provide kinetic stabilization, but dynamic and structural information on those contacts is yet limited. In this study we have accessed the metalogermylenium cation [(PMe_2_Ar^Dipp2^)AuGe(Ar^Dipp2^)Cl]^+^ (**4^+^**) (Ar^Dipp2^=C_6_H_3_‐2,6‐(C_6_H_3_‐2,6‐*i*Pr_2_)_2_) that has been structurally characterized with three different non‐coordinating counter anions. These studies provide for the first time dynamic information about the conformational rearrangement that characterizes π‐arene bonding thorough a series of X‐ray diffraction structural snapshots. Computational studies reveal the weak character of the π‐arene bonding (ca. 2 kcal mol^−1^) that can be described as the donation from a π_C=C_ bond toward the empty p valence orbital of germanium.

## Graphical abstract

**Gold for the soul**: These studies provide, for the first time, dynamic information about the conformational rearrangement that characterizes π‐arene bonding thorough a series of X‐ray diffraction structural snapshots of gold metalogermylenium cation complexes with varying counter anions.

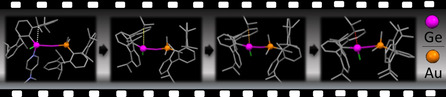

Heavier group 14 element cations have attracted great attention due to their remarkable reactivity and catalytic potential.^[1]^ The quest for base‐free heavier analogs of carbenium cations, R_3_E^+^ (E=Si, Ge, Sn, Pb), has revealed that π‐type interactions are crucial for their stabilization (**A**–**C** in Figure 1).^[2]^ The same applies to the more exotic low‐coordinate tetrylium‐ylidene cations (RE:^+^; **d**–**F** in Figure 1).[^2d^, ^3^] As such, terphenyl (2,6‐diarylphenyl) substituents have become one of the preferred choices to access such highly electrophilic species. The lateral rings of terphenyl moieties can pacify the electron deficiency of the tetrel center while providing steric shielding and preventing the approach of external nucleophiles. In some cases, these interactions may even evolve into highly unusual transformations that involve one of the flanking aryl rings.^[4^—^7]^ Weaker intramolecular π‐arene interactions characterized by longer E⋅⋅⋅C_aryl_ contacts may also be identified in neutral tetrylenes^[8]^ or base‐stabilized tetrylenium cations.^[9]^

**Figure 1:**
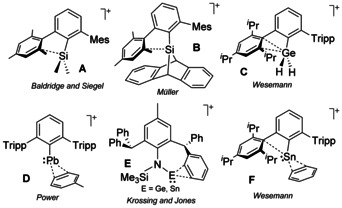
Representative examples of structurally characterized π‐interactions in group 14 cations (Mes=C_6_H_2_‐2,4,6‐Me_3_; Tripp=C_6_H2‐2,4,6‐*i*Pr_3_).

Obtaining a better understanding of the factors governing these interactions is key for further advances in the area and for the discovery of novel transformations, particularly considering that related interactions are crucial to the high activities of transition metal catalysts bearing Buchwald‐type phosphines.^[10]^ With this aim in mind we have targeted systems whose electrophilicities lie between those of R_3_E^+^ cations (strong or moderate π‐interactions) and those of R_2_E: species (weak or negligible π‐contacts). To do so, we decided to enhance the electrophilicity of neutral germylene [Ar^Dipp2^GeCl]_2_ (**1**) (Ar^Dipp2^=C_6_H_3_‐2,6‐(C_6_H_3_‐2,6‐*i*Pr_2_)_2_)^[11]^ by coordination to cationic gold fragments of the type [(PR_2_Ar’)Au]^+[12]^ (Ar’=terphenyl) and investigate the role and nature of lateral π‐contacts. In addition, this strategy facilitates stereoelectronic tunability at germanium by modifying the ligand bound to gold. In this work, not only have we demonstrated the existence of intramolecular π‐arene bonding to germanium, but we provide the trajectory for its reversible formation by a series of solid‐state structures that represent frozen snapshots of dynamic behavior for the same metallogermylene cation.

The insertion of germylenes into Au‐halide bonds is well‐known,^[13]^ and we recently reported a representative example by reacting GeCl_2_ and [(PMe_2_Ar^Dipp2^)AuCl] (**2‐Cl**).^[14]^ The same reactivity readily takes place with half of dimer [Ar^Dipp2^GeCl]_2_ (**1**) to yield gold germyl [(PMe_2_Ar^Dipp2^)AuGe(Ar^Dipp2^)Cl_2_] (**3**) (Scheme 1), characterized by a ^31^P{^1^H} NMR resonance at 11.7 ppm, shifted to higher frequency relative to **2‐Cl** (*δ* −2.5 ppm). We next targeted chloride abstraction in order to generate an electrophilic germanium center that might be primed to interact with one of the pendant aryl rings of its terphenyl ligand. At the same time, we aimed to examine the effects of stereoelectronic modulation at germanium by using a set of weakly to non‐coordinating anions. First, we attempted to convert gold germyl **3** into **4‐NTf_2_** by salt metathesis with AgNTf_2_ (NTf_2_^−^=triflimidate=[N(SO_2_CF_3_)_2_]^−^). This reaction, however, led us to isolate the unexpected trimetallic compound **5** (Scheme 1). Its molecular formulation was elucidated by X‐ray diffraction studies (Figure 2), revealing silver *η* ^2^‐coordination to one of the pendant rings of Ar^Dipp2^ (*d*_AgC_=2.728(7) and 2.570(7) Å).^[15]^ This type of uncommon structure is proposed to form during halide abstraction processes.^[16]^ However, precipitation of AgCl from **5** was not observed even after prolonged periods of time. Instead, compound **4‐NTf_2_** was successfully prepared by the instantaneous reaction between [Ar^Dipp2^GeCl]_2_ (**1**) with [(PMe_2_Ar^Dipp2^)Au(NTf_2_)] (**2‐NTf_2_**) (Scheme 1).

**Scheme 1:**
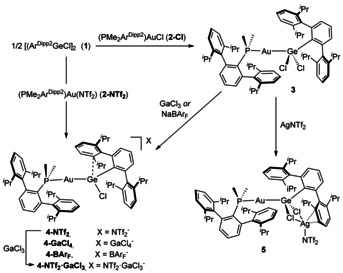
Synthesis of cationic gold germylene compounds of type **4** and trimetallic species **5**. All reactions proceed instantly at room temperature in benzene solution.

**Figure 2:**
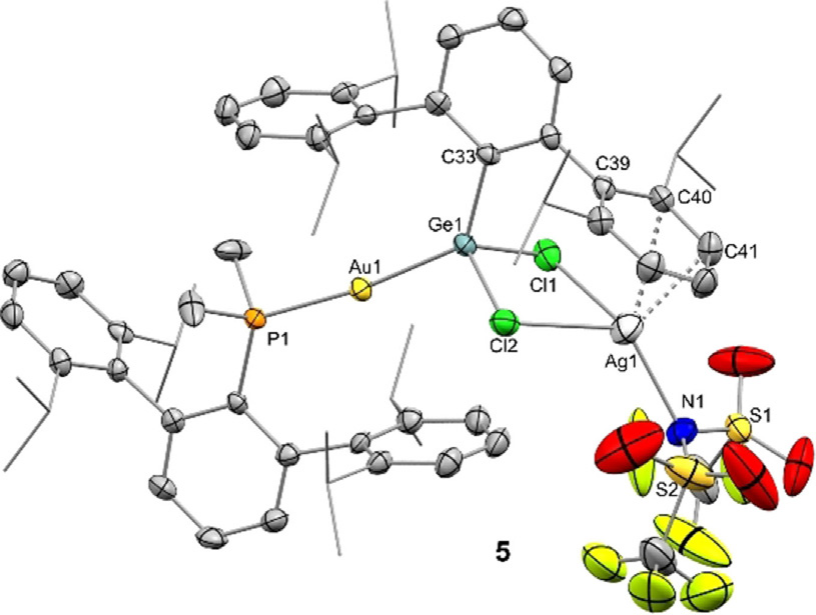
ORTEP diagram of compound **5**; for the sake of clarity hydrogen atoms are excluded and some substituents have been represented in wireframe format, while thermal ellipsoids are set at 50 % probability.

Subsequently, we assessed NaBAr_F_ (BAr_F_=B[C_6_H_3_‐3,5‐(CF3)_2_]_4_^−^) and GaCl_3_ as alternative chloride scavengers (Scheme 1). Their addition to **3** resulted in rapid consumption of gold germyl precursor **3** leading to compounds **4‐BAr_F_** and **4‐GaCl_4_**. Surprisingly, the multinuclear NMR spectra of these compounds not only diverged from those of **4‐NTf_2_** but differed notably from each other. These findings suggest dissimilar molecular formulations in solution for the three related compounds of type **4**, which we attribute to the dissimilar coordinating capacity of the accompanying anions. Diffusion‐Ordered NMR experiments (DOSY) carried out on compounds of type **4** further support the latter assumption (see Supporting Information),^[17]^ evincing that only in **4‐NTf_2_** there is considerable anion binding. In fact, the nature of the counter anion not only affects the NMR spectra. Thus, **4‐NTf_2_** and **4‐GaCl_4_** exhibit moderate stability in solution (*t*_1/2_=14 h at 25 °C), while the fleeting nature of **4‐BAr_F_** (*t*_1/2_=30 min at 25 °C), likely caused by the absence of stabilizing interactions with the BAr_F_^−^ anions, prevented its isolation in analytically pure form. Intriguingly, a fourth related species **4‐NTf_2_⋅GaCl_3_**, characterized by its own set of distinctive multinuclear NMR resonances (Table S1), results from the addition of gallium trichloride to **4‐NTf_2_** (Scheme 1). This novel counter anion is formed due to coordination of the acidic Ga^III^ center to one of the oxygen centers of the triflimidate moiety (see Figure S2).

We were able to grow single crystals of **4‐NTf_2_**, **4‐GaCl_4_** and **4‐NTf_2_⋅GaCl_3_** by either slow solvent evaporation or pentane diffusion into their benzene solutions. To our surprise, these three solid‐state structures revealed a rather peculiar structural variation at cation **4^+^** (Figures 3 and 4). It is important to note that, in contrast with our spectroscopic observations, these solid‐state structures did not contain interactions between the counter anions and gold or germanium (shortest distances >6.5 Å). Even so, the conformation of **4^+^** is markedly altered in the three cases. It is well‐recognized that X‐ray crystallography may provide valuable dynamic information about a precise structural deformation or chemical transformation by means of structure‐correlation approaches.^[18]^ Thus, the structures represented in Figure 3 can be considered as snapshots of the dynamic structural rearrangement that accounts for the reversible formation of π‐arene interactions in group 14 cations (see Figure 4),[^2^, ^3^] which in this case would also involve dynamic exchange of the two lateral aryl rings in solution. The shortest Ge⋅⋅⋅C_aryl_ distances range from 2.492(6) in **4‐NTf_2_** to 2.959(8) Å in **4‐GaCl_4_**, going through an intermediate stage defined by a weak contact of 2.769(7) Å in **4‐NTf_2_⋅GaCl_3_**. Hence, we provide three extreme scenarios for an identical metalogermylene cation **4^+^**, namely those based on a significantly covalent Ge‐C_aryl_ bond (**4‐NTf_2_**), a weak π‐arene interaction (**4‐NTf_2_⋅GaCl_3_**) and a non‐interacting ‘naked’ germanium atom (**4‐GaCl_4_**). Our computational studies (see below) support the existence of the proposed bonding schemes, which result from the combination of minor structural energy differences compensated for by solid‐state effects (counter anion crystal packing; see Figure S5).

**Figure 3:**
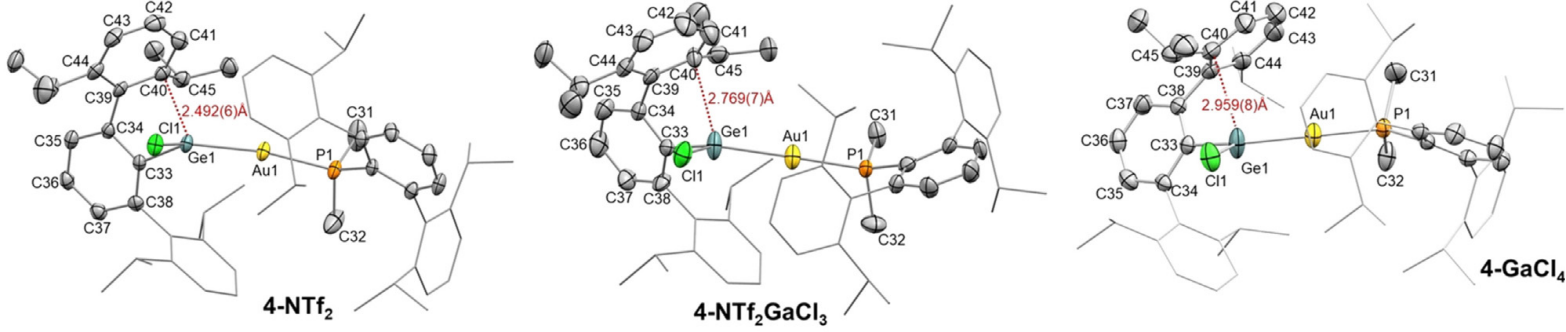
ORTEP diagrams of compounds **4‐NTf_2_**, **4‐NTf_2_⋅GaCl_3_** and **4‐GaCl_4_**; for the sake of clarity hydrogen atoms, solvent molecules and counter anions are excluded and some substituents have been represented in wireframe format, while thermal ellipsoids are set at 50 % probability.

**Figure 4:**
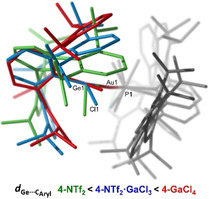
Superimposed representation of X‐ray diffraction structures of cationic parts of **4‐NTf_2_** (green), **4‐NTf_2_⋅GaCl_3_** (blue) and **4‐GaCl_4_** (red) highlighting the structural dynamic rearrangement of the Ge‐bound terphenyl moiety.

In the structure of **4‐NTf_2_** the germanium atom deviates from a symmetrical position relative to its terphenyl substituent (C34‐C33‐Ge1, 111.9(2)°; C38‐C33‐Ge1, 125.5(2)°). This distortion is required to accommodate bonding with the carbon atom at the *ortho* position of a pendant aryl ring, and sheds light on the electrophilic character of the germanium atom. As a consequence of this bonding interaction, the corresponding isopropyl group is bent out from the aryl plane (*d*_C45‐C Aryl plane_=0.583 Å) due to pyramidalization at the quaternary carbon. The germanium center is also pyramidal, as reflected by the sum of covalent bond angles around Ge (352.4(1)°). As expected, the C−C bonds involving the interacting aryl carbon are elongated (C39−C43 1.433(4); C43−C44 1.413(4) Å). The dihedral angle between the coordinating and central aryl rings accounts for 55.02°, a considerable deviation from the almost ideal 90° that is observed for the non‐interacting pendant ring (84.0°). These structural features are comparable to previously reported group 14 cations stabilized by strong π‐type interactions (Figure 1).[^2^, ^3^] As mentioned previously, this situation markedly contrasts with that of the same cation crystallized as **4‐GaCl_4_**, where there is no apparent Ge⋅⋅⋅C_aryl_ interaction. Finally, the structural parameters of **4‐NTf_2_⋅GaCl_3_** lie between the two extreme scenarios found in **4‐NTf_2_** and **4‐GaCl_4_** (see Table 1).

**Table 1:** Selected structural parameters of cations **4^+^**.

Structure	**4‐NTf_2_**	**4‐NTf_2_⋅GaCl_3_**	**4‐GaCl_4_**
Ge1−C33 [Å]	1.952(3)	1.948(9)	1.921(6)
Ge1−Au1 [Å]	2.3941(3)	2.4104(12)	2.3612(7)
Ge1−Cl1 [Å]	2.188(1)	2.166(3)	2.138(2)
C34‐C33‐Ge [°]	111.9(2)	115.3(6)	114.6(4)
C38‐C33‐Ge [°]	125.5(2)	123.3(7)	122.3(4)
Ge⋅⋅⋅C_Aryl_ [Å]	2.492(6)	2.769(7)	2.959(8)
^*i*^Pr bent [Å]^[a]^	0.583	0.426	0.309
Σ_Ge angles_ [°]^[b]^	352.4(1)	356.8(1)	359.8(1)
*d*_C39−C40_ [Å]	1.433(4)	1.426(13)	1.425(10)
*d*_C40−C41_ [Å]	1.413(4)	1.413(4)	1.381(10)
Ar‐Ar [°]^[c]^	55.02	63.34	74.43

[a] Distance from C_45_ (tertiary ^*i*^Pr‐carbon at the interacting aryl) out of the aryl plane. [b] Sum of the three covalent angles involving germanium. [c] Dihedral angle between central and interacting aryl rings.

We have examined the differences in energy of the DFT‐calculated geometries of cation **4^+^** as a function of the shortest Ge⋅⋅⋅C_aryl_ distance in benzene.^[19]^ As anticipated, this difference is small, spanning 2.2 kcal mol^−1^ in the range of 2.4–3.3 Å (Figure S6). Free optimization of the structure derived from **4‐NTf_2_** (the counter anion was omitted) afforded a local minimum, **4 s^+^**, with a shortest Ge⋅⋅⋅C_aryl_ distance of 2.40 Å. On the other hand, the calculations failed to reproduce the X‐ray structures with longer Ge⋅⋅⋅C_aryl_ distances, collapsing to geometries with shorter distances, which suggests a role for intermolecular interactions in the stabilization of these species. When the shortest Ge⋅⋅⋅C_aryl_ distances were fixed to the experimental values of 2.769 (**4 i^+^**) and 2.959 Å (**4 l^+^**), the calculated energies (Δ*ZPE*_benzene_) were 0.99 and 2.27 kcal mol^−1^ respectively, relative to **4 s^+^**.

Electron density (AIM) analysis^[20]^ of the calculated species revealed bond critical points, *bcp*s, and bond paths connecting the expected Ge and C_aryl_ atoms of **4 s^+^** (*ρ*_b_=0.049 a.u.; Figure 5) and **4 i^+^** only, although in the latter case low electron density (*ρ*_b_=0.026 a.u.) and high ellipticity (*ϵ*_b_=1.279) at the *bcp* indicate an unstable critical point. Also, localized orbital (NBO)^[21]^ analysis supports significant π‐arene interaction with the electrophilic germanium in **4 s^+^**. Thus, one π_C=C_ orbital of the aryl ring featuring the shortest Ge⋅⋅⋅C_aryl_ distance is delocalized over one vacant, mostly p, orbital on the Ge, with the corresponding natural localized molecular orbital (NLMO) having 8.3 % Ge character. The analogous NLMOs for **4 i^+^** and **4 l^+^** have 4.2 % and 2.06 % Ge character, respectively (Figure S7).^[22]^

**Figure 5:**
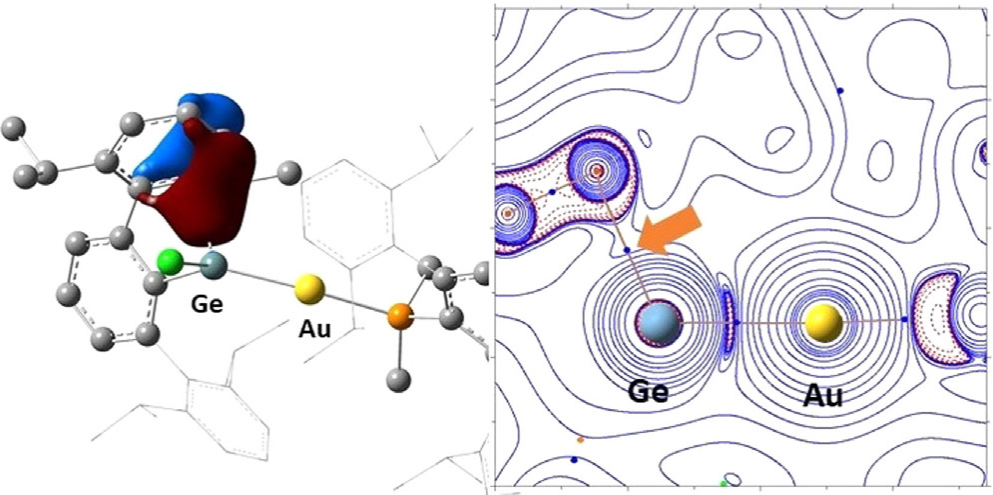
DFT‐optimized geometry of **4 s^+^** with the NLMO for the π_C=C_→*p*_Ge_ interaction (left) and representative bcps and bond paths of its electron density, ***ρ***(r), overlaying the Laplacian ∇^2^ ***ρ***(r) (right).

For the sake of completeness, we decided to explore the reaction of cations **4^+^** with a common Lewis base (4‐dimethylamino pyridine, DMAP) to quench the electrophilicity of the germanium site. As expected, all compounds of type **4** rapidly evolve to the same gold germylene adduct **6** upon DMAP addition (Scheme 2), which reflected in rapid discoloration from intense orange to colorless solutions. The structure of **6** was corroborated by X‐ray diffraction studies (see Figure S4), revealing that the Au−Ge bond remains intact. Conceptually, this structure can be viewed as one step further in the dynamic motion represented by the discussed conformational rearrangement of the interacting terphenyl moiety. Specifically, the germanium site in **6** does not exhibit any hint of weak contacts with the flanking aryl rings of the terphenyl substituents (i.e., shortest Ge⋅⋅⋅C_Aryl_ distance of 3.75 Å and no isopropyl bending from the aryl plane).

**Scheme 2:**
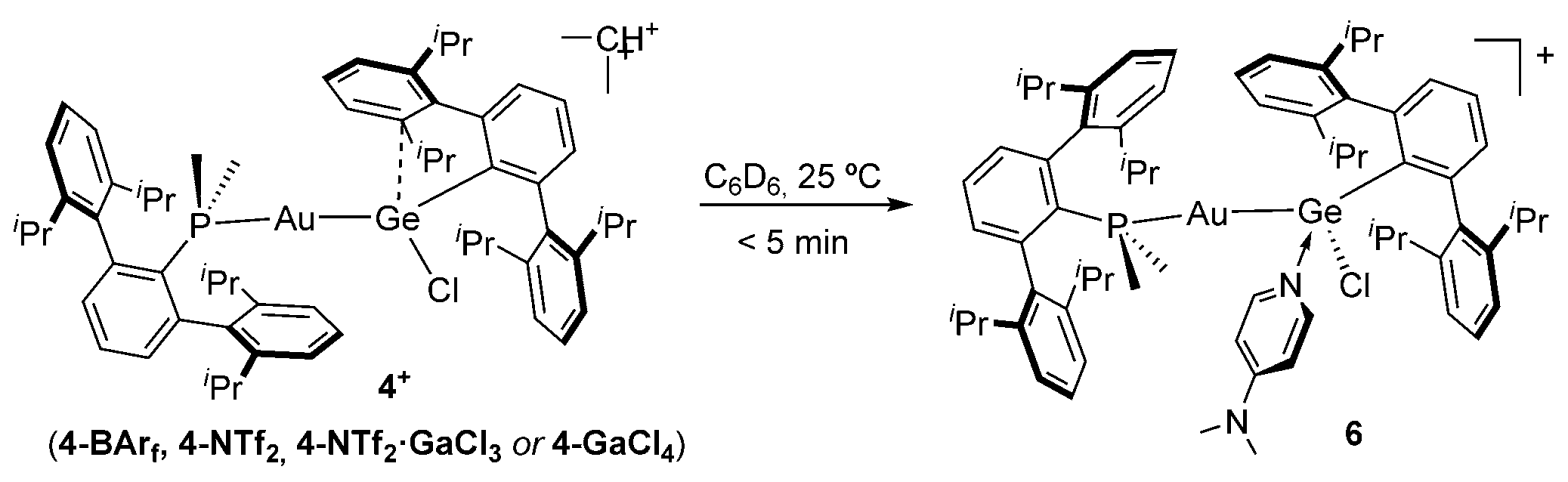
Reaction of cations **4^+^** with Lewis bases towards adducts **6**.

In summary, our studies provide a series of structural snapshots for the dynamic stabilization of a germylenium cation by intramolecular π‐arene interactions. Dynamic information of this kind has so far been elusive due to the weakness of the investigated interaction (ca. 2 kcal mol^−1^), the high reactivity of group 14 cations and their limited solubility. In fact, our results suggest that these contacts may have often been overseen even in neutral tetrylene compounds, despite having an important role to stabilize reactive intermediates as occurs in prominent transition metal catalysts. In addition to London dispersion forces,^[23]^ these interactions likely contribute to the long‐standing success of terphenyl groups and related motifs to stabilize low‐valent main group compounds. Besides, it is important to remark that dynamic information from structure‐correlation methods is typically acquired by ligand or metal modification, while herein this input is gathered from a single compound of interest with no chemical alteration, and only results from crystal packing forces involving non‐coordinating anions. Finally, we evidence that incorporating an electrophilic gold fragment (as a Z‐type ligand) to neutral tetrylenes gives access to electrophilic group 14 species of enhanced reactivity. The potential of those for bond activation and catalysis is currently under exploration.

## Experimental

Full details of synthesis, characterisation, and computational studies can be found in the Supporting Information.

Deposition Numbers 2013214 (**4‐NTf_2_**), 2013219 (**4‐NTf_2_⋅GaCl_3_**), 2013217 (**4‐GaCl_4_**). 2013216 (**3**), 2013215 (**5**) and 2013218 (**6**) contain the supplementary crystallographic data for this paper. These data are provided free of charge by the joint Cambridge Crystallographic Data Centre and Fachinformationszentrum Karlsruhe Access Structures service www.ccdc.cam.ac.uk/structures.

## Conflict of interest

The authors declare no conflict of interest.

## Supporting information

As a service to our authors and readers, this journal provides supporting information supplied by the authors. Such materials are peer reviewed and may be re‐organized for online delivery, but are not copy‐edited or typeset. Technical support issues arising from supporting information (other than missing files) should be addressed to the authors.

Supplementary

## References

[1a] V. S. V. S. N. Swamy , S. Pal , S. Khan , S. S. Sen , Dalton Trans. 2015, 44, 12903–12923; 26084389 10.1039/c5dt01912e

[1b] T. A. Engesser , M. A. Lichtenthaler , M. Schleep , I. Krossing , Chem. Soc. Rev. 2016, 45, 789–899; 26612538 10.1039/c5cs00672d PMC4758321

[1c] H. Fang , Z. Wang , X. Fu , Coord. Chem. Rev. 2017, 344, 214–237.

[2a] S. Duttwyler , Q.-Q. Do , A. Linden , K. K. Baldridge , J. S. Siegel , Angew. Chem. Int. Ed. 2008, 47, 1719–1722; 10.1002/anie.200705291 18205154

[2b] P. Romanato , S. Duttwyler , A. Linden , K. K. Baldridge , J. S. Siegel , J. Am. Chem. Soc. 2011, 133, 11844–11846; 21766853 10.1021/ja2040392

[2c] C. Gerdes , W. Saak , D. Haase , T. Müller , J. Am. Chem. Soc. 2013, 135, 10353–10361; 23786334 10.1021/ja400306h

[2d] F. Diab , F. S. W. Aicher , C. P. Sindlinger , K. Eichele , H. Schubert , L. Wesemann , Chem. Eur. J. 2019, 25, 4426–4434. 30706972 10.1002/chem.201805770

[3a] S. Hino , M. Brynda , A. D. Philips , P. P. Power , Angew. Chem. Int. Ed. 2004, 43, 2655–2658; 10.1002/anie.200353365 18629981

[3b] J. Li , C. Schenk , F. Winter , H. Scherer , N. Trapp , A. Higelin , S. Keller , R. Pottgen , I. Krossing , C. Jones , Angew. Chem. Int. Ed. 2012, 51, 9557–9561; 10.1002/anie.201204601 22936618

[3c] A. Hinz , Chem. Eur. J. 2019, 25, 7843–7846. 30986324 10.1002/chem.201901573

[4a] C. Cui , M. M. Olmstead , P. P. Power , J. Am. Chem. Soc. 2004, 126, 5062–5063; 15099077 10.1021/ja0318481

[4b] C. Cui , M. M. Olmstead , J. C. Fettinger , G. H. Spikes , P. P. Power , J. Am. Chem. Soc. 2005, 127, 17530–17541; 16332105 10.1021/ja055372s

[4c] N. Y. Tashkandi , L. C. Pavelka , C. A. Caputo , P. D. Boyle , P. P. Power , K. M. Baines , Dalton Trans. 2016, 45, 7226–7230. 27064526 10.1039/c6dt01015f

[5] S. Wang , L. Tao , T. A. Stich , M. M. Olmstead , R. D. Britt , P. P. Power , Inorg. Chem. 2017, 56, 14596–14604. 29130691 10.1021/acs.inorgchem.7b02413

[6] D. Raiser , C. P. Sindlinger , H. Schubert , L. Wesemann , Angew. Chem. Int. Ed. 2020, 59, 3151–3155; 10.1002/anie.201914608 PMC7028040 31804742

[7] W. Setaka , K. Hirai , H. Tomioka , K. Sakamotoy , M. Kira , Chem. Commun. 2008, 6558–6560. 10.1039/b814801e 19057778

[8a] M. Usher , A. V. Protchenko , A. Rit , J. Campos , E. L. Kolychev , R. Tirfoin , S. Aldridge , Chem. Eur. J. 2016, 22, 11685–11698; 27381647 10.1002/chem.201601840

[8b] M. L. McCrea-Hendrick , S. Wang , K. L. Gullett , J. C. Fettinger , P. P. Power , Organometallics 2017, 36, 3799;

[8c] K. Izod , P. Evans , P. G. Waddell , Chem. Commun. 2018, 54, 2526–2529. 10.1039/c7cc09564c 29461554

[9a] C. P. Sindlinger , F. S. W. Aicher , L. Wesemann , Inorg. Chem. 2017, 56, 548–560; 27977148 10.1021/acs.inorgchem.6b02377

[9b] C. P. Sindlinger , F. S. W. Aicher , H. Schubert , L. Wesemann , Angew. Chem. Int. Ed. 2017, 56, 2198–2202; 10.1002/anie.201610254 28097796

[10a] C. A. Fleckenstein , H. Plenio , Chem. Soc. Rev. 2010, 39, 694–711; 20111788 10.1039/b903646f

[10b] D. S. Surry , S. L. Buchwald , Chem. Sci. 2011, 2, 27–50. 22432049 10.1039/C0SC00331J PMC3306613

[11] L. Pu , A. D. Phillips , A. F. Richards , M. Stender , R. S. Simons , M. M. Olmstead , P. P. Power , J. Am. Chem. Soc. 2003, 125, 11626–11636. 13129367 10.1021/ja035711m

[12] J. Campos , J. Am. Chem. Soc. 2017, 139, 2944–2947. 28186739 10.1021/jacs.7b00491

[13a] J. A. Cabeza , J. M. Fernández-Colinas , P. García-Álvarez , D. Polo , Inorg. Chem. 2012, 51, 3896–3903; 22375851 10.1021/ic3001575

[13b] A. Bauer , H. Schmidbaur , J. Am. Chem. Soc. 1996, 118, 5324–5325.

[14] N. Hidalgo , S. Bajo , J. J. Moreno , C. Navarro-Gilabert , B. Mercado , J. Campos , Dalton Trans. 2019, 48, 9127–9138. 31139809 10.1039/c9dt00702d

[15] X. Wang , Y. Peng , M. M. Olmstead , A. Hope , P. P. Power , J. Am. Chem. Soc. 2010, 132, 13150–13151. 20809567 10.1021/ja1051236

[16a] D. Carmona , F. Viguri , F. J. Lahoz , L. A. Oro , Inorg. Chem. 2002, 41, 2385–2388; 11978103 10.1021/ic011217c

[16b] S. G. Weber , F. Rominger , B. F. Straub , Eur. J. Inorg. Chem. 2012, 2863–2867;

[16c] G. Sipos , P. Gao , D. Foster , B. W. Skelton , A. N. Sobolev , R. Dorta , Organometallics 2017, 36, 801–817.

[17] A specific discussion on the solution dynamics of compounds **4** based on NMR studies has been incorporated into the Supporting Information (Section 2, Table S1 and Figure S1).

[18a] H. B. Bürgi , J. D. Dunitz , Acc. Chem. Res. 1983, 16, 153–161;

[18b] D. Scheschkewitz , H. Amii , H. Gornitzka , W. W. Schoeller , D. Bourissou , G. Bertrand , Angew. Chem. Int. Ed. 2004, 43, 585–587; 10.1002/anie.200352944 14743410

[18c] O. Ekkert , A. J. P. White , M. R. Crimmin , Angew. Chem. Int. Ed. 2016, 55, 16031–16034; 10.1002/anie.201608599 27879025

[19a] Geometry optimizations performed at the DFT, SMD(benzene)-PBE0-D3/6–31 g(d,p)+SDD) level with the Gaussian 09 software;

[19b] Gaussian 09, Revisions B.01 and E.01; Gaussian, Inc.: 1011 Wallingford CT, **2010** See the Supporting Information for further details.

[20a] R. F. W. Bader , Atoms in Molecules: A Quantum Theory, Oxford University Press, Oxford, 1995;

[20b] The extended wavefunction.wfx and NBO.47 files were calculated at the DFT, SMD(benzene)-PBE0-D3/def-2TZVP level;

[20c] Wavefunction analysis were performed with the Multifwn code: Multiwfn, v. 6.0. http://sobereva.com/multiwfn/.

[21] E. D. Glendening , C. R. Landis , F. Weinhold , J. Comput. Chem. 2013, 34, 1429–1437. 23483590 10.1002/jcc.23266

[22] The LUMO of **4 l^+^** is an almost pure p orbital of the Ge atom, which further supports the notion of a non-interacting germanium atom.

[23] D. J. Liptrot , P. P. Power , Nat. Rev. Chem. 2017, 1, 0004.

