# Supplementary material for: Structural Snapshots of π‐Arene Bonding in a Gold Germylene Cation
Source: Chemistry. 2020 Nov 9;26(67):15519–23. doi: 10.1002/chem.202004566 (PMC7756455; doi:10.1002/chem.202004566)
Supplement: Supplementary file 1 — Supplementary [file CHEM-26-15519-s001.pdf]

Chemistry–A European Journal

Supporting Information

Structural Snapshots of π-Arene Bonding in a Gold Germylene Cation

Sonia Bajo, María M. Alcaide, Joaquín López-Serrano, and Jesús Campos*[a]

1. Synthesis and characterization of new compounds	S2
2. Solution NMR analysis of compounds of type **4**	S10
3. X-Ray structural characterization of new compounds.	S14
4. Computational details	S20
5. NMR spectra of new compounds	S26
6. References	S36

1. Synthesis and characterization of new compounds

General considerations. All manipulations were carried out using standard Schlenk and glove-box techniques, under an atmosphere of argon and of high purity nitrogen, respectively. All solvents were dried, stored over 4 Å molecular sieves, and degassed prior to use. *N*-pentane (C_5H_{12}) were distilled under nitrogen over sodium. [D6]Benzene was distilled under argon over sodium/benzophenone. Compounds **1**,[1] **2**[2] and $NaBAr_F$[3] were prepared as described previously. Other chemicals were commercially available and used as received. Solution NMR spectra were recorded on Bruker AMX-300, DRX-400 and DRX-500 spectrometers. Spectra were referenced to external SiMe4 (δ: 0 ppm) using the residual proton solvent peaks as internal standards (^1H NMR experiments), or the characteristic resonances of the solvent nuclei (^{13}C NMR experiments), while ^{31}P was referenced to H_3PO_4. Spectral assignments were made by routine one- and two-dimensional NMR experiments (^1H, $^{13}C\{^1H\}$, $^{31}P\{^1H\}$, COSY, NOESY, HSQC and HMBC) where appropriate. For elemental analyses a LECO TruSpec CHN elementary analyzer, was utilized.

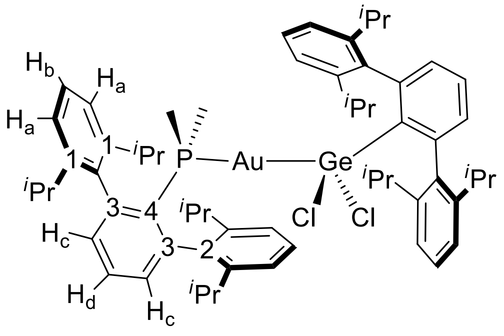

Compound 3. In a NMR tube, to a solution of [$PMe_2Ar^{Dipp_2}$]AuCl (**2-Cl**; 30 mg, 0.04 mmol) in C_6D_6 (0.5 mL) was added $(Ar'GeCl)_2$ (Ar' = C_6H_3-2,6-$Dipp_2$, Dipp = C_6H_3-2,6-$^i Pr_2$) (22 mg, 0.02 mmol). The initial orange solution became colorless instantaneously. Compound **3** can be crystallized by slow evaporation of the C_6D_6 solution (27 mg, 52%).

Anal. Calcd for $C_{62}H_{80}AuCl_2GeP$: C, 62.2; H, 6.7. Found: C, 62.4; H, 6.6.

^{1}H NMR (400 MHz, C_6D_6, 298 K): δ 7.38 (t, $^3J_{HH}$ = 7.6 Hz, 2H, H$_b$), 7.19 (d, $^3J_{HH}$ = 7.6 Hz, 4H, H$_a$), 7.08 (m, 9H, CH), 6.93 (overlapping signals, 3H, H$_c$ + H$_d$), 3.14 (sept, $^3J_{HH}$ = 6.6 Hz, 4H, C*H*(CH$_3$)$_2$ Ge), 2.52 (sept, $^3J_{HH}$ = 6.6 Hz, 4H, C*H*(CH$_3$)$_2$ Au), 1.46 (d, $^3J_{HH}$ = 6.6 Hz, 12H, CH(C*H*$_3$)$_2$ Ge), 1.33 (d, $^3J_{HH}$ = 6.6 Hz, 12H, CH(C*H*$_3$)$_2$ Au), 1.10 (d, $^2J_{HP}$ = 8.7 Hz, 6H, PMe$_2$), 1.05 (d, $^3J_{HH}$ = 6.6 Hz, 12H, CH(C*H*$_3$)$_2$ Ge), 0.88 (d, $^3J_{HH}$ = 6.6 Hz, 12H, CH(C*H*$_3$)$_2$ Au).

^{31}P{^{1}H} NMR (161.98 MHz, C_6D_6, 298 K): δ 11.7 (s).

^{13}C{^{1}H} NMR (100.63 MHz, C_6D_6, 298 K): δ 148.3 (d, $^2J_{CP}$ = 25 Hz, C$_3$), 147.4 (s, *o*-Dipp Ge), 145.7 (s, C$_1$), 145.6 (s, *i*-C$_6$H$_3$ Ge), 145.5 (s, C$_q$ Ge), 140.4 (s, *i*-Dipp Ge), 137.6 (d, $^3J_{CP}$ = 5 Hz, C$_2$), 132.3 (d, $^3J_{CP}$ = 7 Hz, CH$_c$), 131.3 (s, CH Ge), 130.6 (d, $^1J_{CP}$ = 42 Hz, C$_4$), 130.2 (s, CH$_b$), 128.9 (s, CH$_d$), 124.0 (s, CH$_a$), 122.5 (s, *m*-Dipp Ge), 31.2 (s, *C*H(CH$_3$)$_2$ Ge), 30.9 (s, *C*H(CH$_3$)$_2$ Au), 25.2 (s, CH(*C*H$_3$)$_2$ Ge + Au), 23.6 (s, CH(*C*H$_3$)$_2$ Ge), 22.8 (s, CH(*C*H$_3$)$_2$ Au), 15.8 (d, $^1J_{CP}$ = 30 Hz, PMe$_2$).

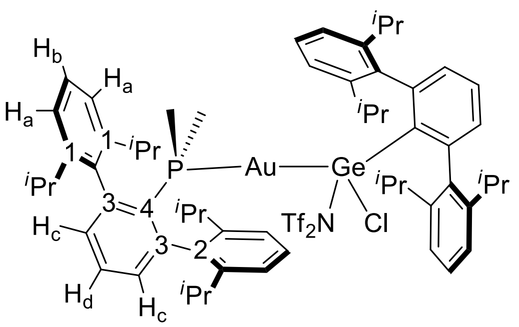

Compound 4-NTf$_2$. In a NMR tube, to a solution of [PMe$_2$Ar^{Dipp_2}]AuNTf$_2$ (**2-NTf$_2$**; 20 mg, 0.02 mmol) in C_6D_6 (0.5 mL) was added (Ar'GeCl)$_2$ (11 mg, 0.01 mmol). The initial orange solution became colorless instantaneously. Compound **4-NTf$_2$** can be crystallized by slow evaporation of the C_6D_6 solution (19 mg, 63%).

Anal. Calcd for $C_{64}H_{80}AuClF_6GeNO_4PS_2$: C, 53.3; H, 5.6; S, 4.4. Found: C, 53.5; H, 5.7; S, 4.3.

^{1}H NMR (400 MHz, tol-d_8, 298 K): δ 7.25 (m, 3H, CH), 7.14 (m, 10H, CH), 7.00 (m, 5H, CH), 2.84 (sept, $^3J_{HH}$ = 6.4 Hz, 4H, C*H*(CH$_3$)$_2$ Ge), 2.52 (sept, $^3J_{HH}$ = 6.4 Hz, 4H, C*H*(CH$_3$)$_2$ Au), 1.37 (d, $^3J_{HH}$ = 6.4 Hz, 12H, CH(C*H*$_3$)$_2$ Ge), 1.34 (d, $^3J_{HH}$ = 6.4 Hz, 12H, CH(C*H*$_3$)$_2$ Au), 1.21 (d, $^2J_{HP}$ = 9.5 Hz, 6H, PMe$_2$), 1.02 (d, $^3J_{HH}$ = 6.4 Hz, 12H, CH(C*H*$_3$)$_2$ Ge), 0.93 (d, $^3J_{HH}$ = 6.4 Hz, 12H, CH(C*H*$_3$)$_2$ Au).

^{31}P{^{1}H} NMR (161.98 MHz, tol-d_8, 298 K): δ 10.0 (s).

^{13}C{^{1}H} NMR (100.63 MHz, tol-d_8, 298 K): δ 148.1 (d, $^2J_{CP}$ = 25 Hz, C$_3$), 146.9 (s, *o*-Dipp Ge), 146.0 (s, C$_1$), 145.2 (s, C$_q$ Ge), 138.4 (s, *i*-Dipp Ge), 132.5 (d, $^3J_{CP}$ = 7 Hz, CH$_c$), 131.6 (s, CH), 129.7 (s, CH), 129.3 (s, CH), 123.9 (s, CH), 123.7 (s, CH), 31.2 (s, *C*H(CH$_3$)$_2$ Ge), 31.0 (s, *C*H(CH$_3$)$_2$ Au), 25.3 (s, CH(*C*H$_3$)$_2$ Ge), 25.2 (s, CH(*C*H$_3$)$_2$ Au), 23.3 (s, CH(*C*H$_3$)$_2$ Ge), 23.0 (s, CH(*C*H$_3$)$_2$ Au), 16.3 (d, $^1J_{CP}$ = 32 Hz, PMe$_2$).

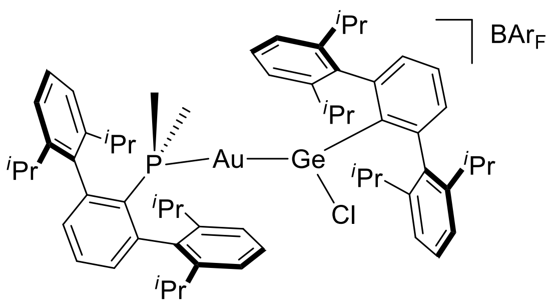

Compound 4-BAr$_F$. In a NMR tube, to a solution of [$PMe_2Ar^{Dipp_2}$]AuCl (**2-Cl;** 20 mg, 0.03 mmol) in C_6D_6 (0.5 mL) was added (Ar'GeCl)$_2$ (15 mg, 0.015 mmol). After 5 min of shacking NaBAr$_F$ (26 mg, 0.03 mmol) was subsequently added to provide **4-BAr$_F$** instantaneously (100% NMR yield). Rapid decomposition and low solubility at lower temperatures prevented the acquisition of reliable $^{13}C\{^1H\}$ NMR data, which were obtained for other related **4**$^+$ cations.

^{1}H NMR (500 MHz, C_6D_6, 298 K): δ 8.54 (s, 8H, *o*-Ar), 7.82 (s, 4H, *p*-Ar), 7.38 (m, 2H, CH), 7.27 (m, 6H, CH), 7.05 (m, 10H, CH), 2.96 (sept, $^3J_{HH}$ = 6.0 Hz, 4H, C*H*(CH$_3$)$_2$ Ge), 2.62 (sept, $^3J_{HH}$ = 6.0 Hz, 4H, C*H*(CH$_3$)$_2$ Au), 1.42 (d, $^3J_{HH}$ = 6.0 Hz, 12H, CH(C*H*$_3$)$_2$ Ge), 1.36 (d, $^3J_{HH}$ = 6.0 Hz, 12H, CH(C*H*$_3$)$_2$ Au), 1.22 (d, $^2J_{HP}$ = 8.5 Hz, 6H, PMe$_2$), 1.05 (br, 24H, CH(C*H*$_3$)$_2$ Ge + Au).

$^{31}P\{^1H\}$ NMR (202.46 MHz, C_6D_6, 298 K): δ 9.8 (s).

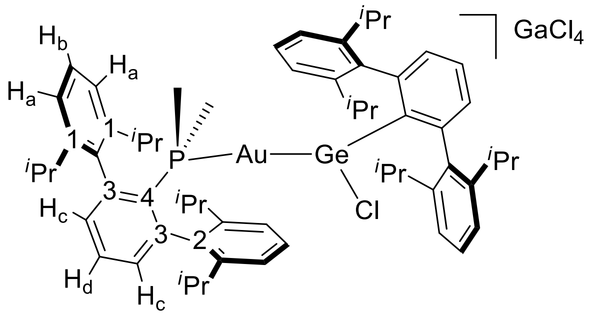

Compound 4-GaCl$_4$. In a NMR tube, a solution of [PMe$_2$Ar^{Dipp_2}]AuCl (**2-Cl;** 30 mg, 0.04 mmol) in C$_6$D$_6$ (0.5 mL) was added (Ar'GeCl)$_2$ (22 mg, 0.02 mmol) and GaCl$_3$ (7.6 mg, 0.04 mmol). The initial orange solution became a colorless mixture instantaneously. Compound **4-GaCl$_4$** can be crystallized by slow diffusion of pentane into a C$_6$D$_6$ solution (2:1 by vol.) (33 mg, 56%).

Anal. Calcd for C$_{62}$H$_{80}$AuCl$_5$GaGeP: C, 54.2; H, 5.9. Found: C, 54.2; H, 6.2.

^{1}H NMR (400 MHz, C$_6$D$_6$, 298 K): δ 7.37 - 6.95 (br, 18H, CH), 2.76 (br, 4H, C*H*(CH$_3$)$_2$), 2.46 (sept, $^3J_{HH}$ = 6.8 Hz, 4H, C*H*(CH$_3$)$_2$), 1.33 (d, $^3J_{HH}$ = 6.8 Hz, 12H, CH(C*H*$_3$)$_2$ Ge), 1.27 (br overlapping signals, 18H, CH(C*H*$_3$)$_2$ Au + PMe$_2$), 0.97 (d, $^3J_{HH}$ = 6.4 Hz, 12H, CH(C*H*$_3$)$_2$ Ge), 0.94 (d, $^3J_{HH}$ = 6.4 Hz, 12H, CH(C*H*$_3$)$_2$ Au).

^{31}P{^{1}H} NMR (161.98 MHz, C$_6$D$_6$, 298 K): δ 5.7 (br).

^{13}C{^{1}H} NMR (100.63 MHz, C$_6$D$_6$, 298 K): δ 157.2 (br, C$_q$), 149.6 (s, C$_q$), 146.1 (s, C$_q$), 145.7 (s, C$_q$), 145.6 (s, C$_q$), 142.8 (s, C$_q$), 138.8 (br, C$_q$), 137.3 (d, $^3J_{CP}$ = 5 Hz, C$_2$), 132.5 (d, $^3J_{CP}$ = 7 Hz, CH$_c$), 131.4 (s, CH), 130.3 (s, CH), 129.5 (s, CH), 129.2 (s, CH), 124.3 (s, CH), 123.7 (s, CH), 122.2 (s, CH), 31.3 (s, *C*H(CH$_3$)$_2$), 31.1 (s, *C*H(CH$_3$)$_2$), 25.7 (s, CH(*C*H$_3$)$_2$), 25.2 (s, CH(*C*H$_3$)$_2$), 23.0 (s, CH(*C*H$_3$)$_2$), 22.8 (s, CH(*C*H$_3$)$_2$), 16.0 (d, $^1J_{CP}$ = 33 Hz, PMe$_2$).

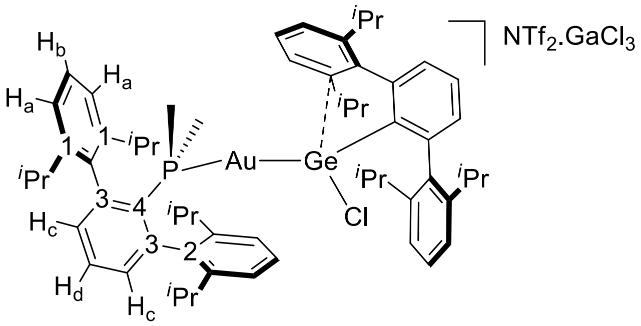

Compound 4-NTf$_2$·GaCl$_3$. In a NMR tube, to a solution of [PMe$_2$Ar^{Dipp_2}]AuNTf$_2$ (**2-NTf$_2$**; 30 mg, 0.03 mmol) in C_6D_6 (0.5 mL) was added (Ar'GeCl)$_2$ (16 mg, 0.016 mmol). After 5 minutes of shacking, GaCl$_3$ (6 mg, 0.03 mmol) was subsequently added. Compound **4-NTf$_2$·GaCl$_3$** can be crystallized by slow diffusion of pentane into a C_6D_6 solution (2:1 by vol.) (25 mg, 48%).

Anal. Calcd for $C_{64}H_{80}AuCl_4F_6GaGeNO_4PS_2$: C, 47.5; H, 5.0; S, 4.0. Found: C, 47.5; H, 5.2 S, 4.3.

^{1}H NMR (400 MHz, C_6D_6, 298 K): δ 7.33 (m, 2H, CH), 7.27 (m, 1H, CH), 7.20 (br, 9H, CH), 7.07 (br, 3H, CH), 7.00 (m, 1H, CH), 6.95 (m, 2H, CH), 2.67 (sept, $^3J_{HH}$ = 6.0 Hz, 4H, C*H*(CH$_3$)$_2$ Ge), 2.41 (sept, $^3J_{HH}$ = 6.8 Hz, 4H, C*H*(CH$_3$)$_2$ Au), 1.28 (overlapping signals, 18H, CH(C*H*$_3$)$_2$ Ge + PMe$_2$), 1.17 (d, $^3J_{HH}$ = 6.8, 12H, CH(C*H*$_3$)$_2$ Au), 0.94 (overlapping signals, 24H, CH(C*H*$_3$)$_2$ Ge + Au).

^{31}P{^{1}H} NMR (161.98 MHz, C_6D_6, 298 K): δ 5.5 (s).

^{13}C{^{1}H} NMR (100.63 MHz, C_6D_6, 298 K): δ 156.9 (d, $^2J_{CP}$ = 18 Hz, C$_3$), 146.5 (s, *o*-Dipp Ge), 146.2 (s, C$_1$), 145.7 (s, *i*-C$_6$H$_3$ Ge),145.6 (s, C$_q$ Ge), 142.8 (s, *i*-Dipp Ge), 138.5 (s, C$_q$ Ge), 137.2 (d, $^3J_{CP}$ = 6 Hz, C$_2$), 132.6 (d, $^3J_{CP}$ = 7 Hz, CH$_c$), 131.7 (s, CH), 131.2 (s, CH), 130.6 (s, CH), 130.4 (s, CH), 129.1 (s, CH), 126.0 (d, $^1J_{CP}$ = 54, C$_4$), 124.3 (s, CH$_a$), 31.3 (s, *C*H(CH$_3$)$_2$), 31.1 (s, *C*H(CH$_3$)$_2$), 25.7 (s, CH(*C*H$_3$)$_2$), 25.1 (s, CH(*C*H$_3$)$_2$), 22.9 (s, CH(*C*H$_3$)$_2$), 22.5 (s, CH(*C*H$_3$)$_2$), 15.9 (d, $^1J_{CP}$ = 34 Hz, PMe$_2$).

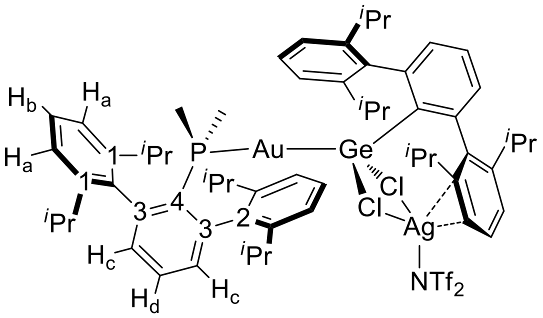

Compound 5. In a NMR tube, to a solution of [PMe$_2$Ar^{Dipp_2}]AuCl (**2-Cl;** 30 mg, 0.04 mmol) in C_6D_6 (0.5 mL) was added (Ar'GeCl)$_2$ (22 mg, 0.02 mmol) and subsequently AgNTf$_2$ (17 mg, 0.04 mmol). The initial orange solution became a colorless mixture instantaneously. Compound **5** can be crystallized by slow diffusion of pentane into a C_6D_6 solution (2:1 by vol.) (36 mg, 53%).

Anal. Calcd for $C_{64}H_{80}AgAuCl_2F_6GeNO_4PS_2$: C, 48.5; H, 5.1; S, 4.0. Found: C, 48.4; H, 5.0; S, 4.3.

^{1}H NMR (400 MHz, C_6D_6, 298 K): δ 7.39 (m, 2H, CH), 7.21 (m, 4H, CH), 7.08 (m, 9H, CH), 6.92 (br, 3H, CH), 3.09 (sept, $^3J_{HH}$ = 5.6 Hz, 4H, C*H*(CH$_3$)$_2$ Ge), 2.51 (sept, $^3J_{HH}$ = 6.4 Hz, 4H, C*H*(CH$_3$)$_2$ Au), 1.44 (d, $^3J_{HH}$ = 5.6 Hz, 12H, CH(C*H*$_3$)$_2$ Ge), 1.33 (d, $^3J_{HH}$ = 6.4 Hz, 12H, CH(C*H*$_3$)$_2$ Au), 1.10 (d, $^2J_{HP}$ = 8.6 Hz, 6H, PMe$_2$), 1.02 (d, $^3J_{HH}$ = 5.6 Hz, 12H, CH(C*H*$_3$)$_2$ Ge), 0.88 (d, $^3J_{HH}$ = 6.4 Hz, 12H, CH(C*H*$_3$)$_2$ Au).

^{31}P{^{1}H} NMR (161.98 MHz, C_6D_6, 298 K): δ 11.2 (s).

^{13}C{^{1}H} NMR (100.63 MHz, C_6D_6, 298 K): δ 148.2 (d, $^2J_{CP}$ = 25 Hz, C$_3$), 147.5 (s, *o*-Dipp Ge), 145.7 (s, C$_1$), 145.6 (s, *i*-C$_6$H$_3$ Ge), 145.4 (s, C$_q$ Ge), 140.4 (s, *i*-Dipp Ge), 137.6 (d, $^3J_{CP}$ = 5 Hz, C$_2$), 132.3 (d, $^3J_{CP}$ = 7 Hz, CH$_c$), 131.3 (s, CH), 130.5 (d, $^1J_{CP}$ = 41 Hz, C$_4$), 130.2 (s, CH), 128.9 (s, CH), 124.0 (s, CH$_a$), 122.4 (s, CH Ge), 31.2 (s, *C*H(CH$_3$)$_2$ Ge), 30.9 (s, *C*H(CH$_3$)$_2$ Au), 25.4 (s, CH(*C*H$_3$)$_2$), 23.5 (s, CH(*C*H$_3$)$_2$), 22.8 (s, CH(*C*H$_3$)$_2$), 15.7 (d, $^1J_{CP}$ = 30 Hz, PMe$_2$).

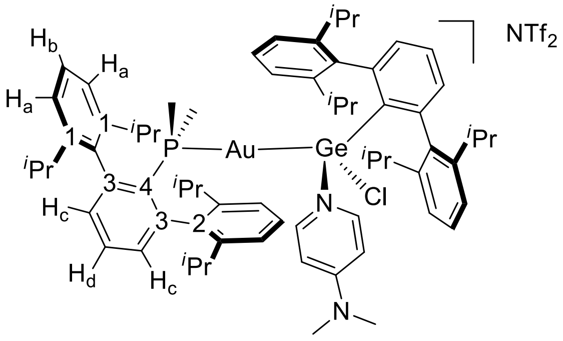

Compound 6. In a NMR tube, to a solution of [$PMe_2Ar^{Dipp_2}$]$AuNTf_2$ (**2-NTf$_2$**; 30 mg, 0.03 mmol) in C_6D_6 (0.5 mL) was added (Ar'GeCl)$_2$ (16 mg, 0.016 mmol) and subsequently 4-Dimethylaminopyridine (4 mg, 0.03 mmol). The initial orange solution became a colorless solution instantaneously. Compound **6** could be crystallized by slow evaporation of a C_6D_6 solution (37 mg, 74%).

Anal. Calcd for $C_{71}H_{90}AuClF_6GeN_3O_4PS_2$: C, 54.5; H, 5.8; S, 4.1. Found: C, 54.2; H, 5.9; S, 4.1.

^{1}H NMR (500 MHz, C_6D_6, 298 K): δ 7.80 (d, $^3J_{HH}$ = 6.5 Hz, 2H, CH_{DMAP}), 7.35 (m, 2H, CH), 7.26 (m, 2H, CH), 7.14-7.06 (overlapping signals, 12H, CH), 6.98 (m, 2H, CH), 6.57 (d, $^3J_{HH}$ = 6.5 Hz, 2H, CH_{DMAP}), 3.11 (sept, $^3J_{HH}$ = 6.4 Hz, 2H, CH(CH$_3$)$_2$), 2.91 (s, 6H, CH_{3DMAP}), 2.82 (sept, $^3J_{HH}$ = 6.4 Hz, 2H, CH(CH$_3$)$_2$), 2.61 (sept, $^3J_{HH}$ = 6.4 Hz, 4H, CH(CH$_3$)$_2$), 1.69 (d, $^2J_{HP}$ = 8.5 Hz, 3H, PMe$_2$), 1.63 (d, $^3J_{HH}$ = 6.4 Hz, 6H, CH(CH_3)$_2$), 1.51 (d, $^3J_{HH}$ = 6.4 Hz, 6H, CH(CH_3)$_2$), 1.38 (d, $^3J_{HH}$ = 6.4 Hz, 6H, CH(CH_3)$_2$), 1.35 (d, $^3J_{HH}$ = 6.4 Hz, 6H, CH(CH_3)$_2$), 1.27 (d, $^2J_{HP}$ = 9.5 Hz, 3H, PMe$_2$), 1.10 (d, $^3J_{HH}$ = 6.4 Hz, 6H, CH(CH_3)$_2$), 1.01 (overlapping signals, 12H, CH(CH_3)$_2$), 0.96 (d, $^3J_{HH}$ = 6.4 Hz, 6H, CH(CH_3)$_2$).

^{31}P{^{1}H} NMR (202.46 MHz, C_6D_6, 298 K): δ 11.0 (s).

^{13}C{^{1}H} NMR (100.63 MHz, C_6D_6, 298 K): δ 155.5 (s, $C_{q\,DMAP}$), 147.2 (s, C_q), 147.0 (s, C_q), 146.2 (s, C_q), 146.0 (s, C_q), 145.6 (s, C_q), 143.5 (s, CH_{DMAP}), 145.2 (s, C_q), 143.4 (s, C_q), 139.0 (s, C_q), 137.7 (d, $^3J_{CP}$ = 5 Hz, C_2), 132.3 (d, $^3J_{CP}$ = 7 Hz, CH_c), 132.1 (s, CH), 131.7 (s, CH), 130.0 (d, $^1J_{CP}$ = 45 Hz, C_4), 129.6 (s, CH), 129.2 (s, CH), 129.1 (s, CH), 124.3 (s, CH), 123.3 (s, CH), 123.0 (s, CH), 122.9 (s, CH), 107.9 (s, CH_{DMAP}), 39.2 (s, Me_{DMAP}), 31.3 (s, CH(CH$_3$)$_2$), 31.1 (s, CH(CH$_3$)$_2$), 30.9 (s, CH(CH$_3$)$_2$), 30.8 (s, CH(CH$_3$)$_2$), 25.4 (s, CH(CH$_3$)$_2$), 25.3 (s, CH(CH$_3$)$_2$), 25.2 (s, CH(CH$_3$)$_2$), 23.4 (s, CH(CH$_3$)$_2$), 23.3 (s, CH(CH$_3$)$_2$), 22.9 (s, CH(CH$_3$)$_2$), 22.8 (s, CH(CH$_3$)$_2$), 17.0 (d, $^1J_{CP}$ = 34 Hz, PMe$_2$), 15.5 (d, $^1J_{CP}$ = 31 Hz, PMe$_2$).

2. Solution NMR analysis of compounds of type 4.

Cation **4**$^+$ has been prepared with four weakly coordinating counteranions, namely BAr_F^-, NTf_2^-, $GaCl_4^-$ and the unusual adduct $NTf_2{\cdot}GaCl_3^-$. X-ray crystallographic analysis for compounds **4-NTf₂**, **4-NTf₂·GaCl₃** and **4-GaCl₄** revealed no close contacts in the solid state between those counteranions and the cationic metalogermylene fragment. The same would be expected for the even less coordinating and more sterically encumbered BAr_F^- anion. Under these circumstances virtually identical multinuclear NMR spectroscopic spectra would be anticipated for the four compounds of type **4**. However, their corresponding ^{1}H, ^{13}C{^{1}H} and ^{31}P{^{1}H} NMR spectra are clearly distinguishable (*vide supra*). Table S1 collects six selected sets of signals for each compound, more precisely four ^{1}H NMR resonances associated to the *iso*-propyl groups of the two terphenyl substituents, one signal corresponding to the methyl groups of the phosphines as well as the corresponding ^{31}P{^{1}H} NMR signals. Exhaustive multinuclear NMR data for these compounds can be found in the previous section, while all relevant spectra are collected in section 5 of this Supporting Information. For clarity, a stacked representation of their ^{1}H NMR spectra is depicted in Figure S1. This representation, as well as the δ values included in Table S1, provides some evidence for the existence of cation-anion interactions, since all other parameters (temperature, solvent or concentration) are strictly kept identical. This is not surprising considering the existence of similar interactions in both transition and main group metal systems. For instance, triflimidate is known to be weakly coordinating, with abundant solid-state structures in which it remained bound to transition[2,4] and main group metals.[5] The same would be expected for the related $[NTf_2{\cdot}GaCl_3]^-$ anionic Lewis pair, though its predicted reduced basicity and increased steric profile may lead to weaker interactions or alternative coordination modes. There are also numerous examples where $GaCl_4^-$ anion interacts with metals,[6] and even for the very weakly coordinating BAr_F^- fragment precedents exist,[7] though the latter would likely be forbidden for a highly congested system as **4**$^+$.

Table S1. Selected NMR spectroscopic data for compounds of type 4 (labels 'Ge' and 'Au' refer to the metal to which the analyzed terphenyl fragment is connected).

Compound	^{1}H, δ C*H*(CH$_3$)$_2$ 'Ge'	^{1}H, δ C*H*(CH$_3$)$_2$ 'Au'	^{1}H, δ CH(C*H*$_3$)$_2$ 'Ge'	^{1}H, δ CH(C*H*$_3$)$_2$ 'Au'	^{1}H, δ PMe$_2$	^{31}P{^{1}H}, δ
**4-NTf$_2$**	2.84	2.52	1.37<br>1.02	1.34<br>0.93	1.21	10.0
**4-BAr$_F$**	2.96	2.62	1.42<br>1.05(br)	1.36<br>1.05(br)	1.22	9.8
**4-GaCl$_4$**	2.76(br)	2.46	1.33<br>0.97	1.27(br)<br>0.94	1.27(br)	5.7 (br)
**4-NTf$_2$·GaCl$_3$**	2.67	2.41	1.28<br>0.94	1.17<br>0.94	1.28	5.5

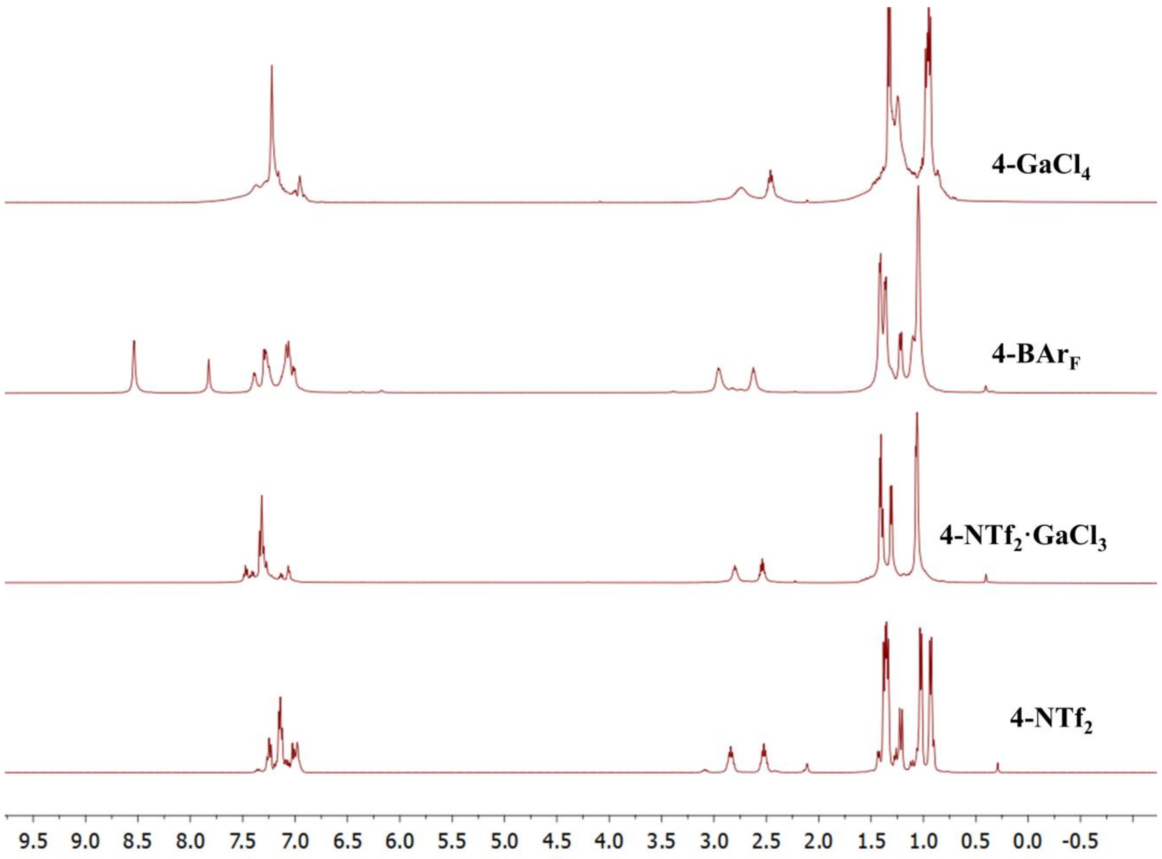

Figure S1. Stacked ^{1}H NMR spectra of compounds of type 4

As stated above, we hypothesize that the dissimilar NMR spectroscopic signals of the four reported compounds of type **4** derived from the existence of counteranion bonding/interactions. Thus, we assume that the data recorded for **4-BAr$_F$** may correspond to the genuine cationic species without anion close contacts, that is, the kind of structures that we have investigated by X-ray diffraction studies (Figures 3 and 4 in the main text), those in which the germanium center interacts with one of the flanking aryl ring of a terphenyl substituent. The absence of cation-anion bonding is consistent with the reduced stability in solution of **4-BAr$_F$** compared to the other analogue species (see main text). Diffusion-Ordered NMR experiments (DOSY) are in agreement with this assumption. In these experiments, if the ion pair diffuse through solution as a single molecular aggregate, the two fragments should lead to identical diffusion coefficients. However, the values calculated from two-dimensional DOSY experiments (0.02 M, C_6D_6, 25 ºC) for compound **4-BAr$_F$** reveal different values associated to the ^{1}H NMR signals due to the BAr$_F$ anion ($2.16\cdot10^{-10}$ $m^2\cdot s^{-1}$) and the metalogermylenium cation ($2.52\cdot10^{-10}$ $m^2\cdot s^{-1}$), suggesting that the two ions do not paired continuously in solution.

On their side, we favor the existence of a Ge—N(SO_2CF_3)$_2$ bond in solution for **4-NTf$_2$**, as supported by computational studies (NTf_2^- coordination seems to be exergonic by 1.02 kcal·mol^{-1} with respect to cation **4$^+$** with a Ge···C$_{Aryl}$ bond and cleavage of the π-arene bonding in this cation raises the energy by *ca.* 2 kcal·mol^{-1}; see main text and section 4 of the Supporting Information). We could not, however, measure appropriate signals by mono and bidimensinoal ^{1}H-^{19}F HOESY NMR correlations, possibly due to the fast relaxation of the fluorine nuclei.[8] Nonetheless, DOSY experiments on **4-NTf$_2$** undertaken under identical conditions to the aforementioned studies provided a diffusion coefficient ($4.76\cdot10^{-10}$ $m^2\cdot s^{-1}$) that doubles that of the BAr$_F$ salt. Faster diffusion rates are associated to neutral species compared to their charged analogues due to the existence of second-sphere solvent shells.[9] Thus, the higher diffusion rate of **4-NTf$_2$** is in accordance with the proposed bonding of the triflimide anion. On the same line, weaker bonding is assumed for **4-GaCl$_4$** and **4-NTf$_2$·GaCl$_3$**, where the measured diffusion coefficients account for $2.87\cdot10^{-10}$ $m^2\cdot s^{-1}$ and $2.63\cdot10^{-10}$ $m^2\cdot s^{-1}$, respectively. Besides, in compound **4-GaCl$_4$** broad ^{1}H and ^{31}P{^{1}H} resonances were collected, providing some evidence of dynamic behavior in solution (though they could not be resolved at low temperature due to poor solubility). As anticipated, the addition of a Lewis base such as DMAP to any of the reported compounds of type **4** (see Scheme 2 in

main text) is sufficient to exclude these interactions leading to **6**, which now exhibits the exact same multinuclear NMR spectra disregarding the nature of the counteranion.

The presence of π-arene bonding with germanium was not obvious at room temperature since a single degenerate structure was recorded by multinuclear NMR spectroscopy. Our attempts to freeze the $Ge\cdots C_{Aryl}$ bond in solution were unsuccessful, partly because of the reduced solubility of **4-BAr$_F$** (as well as of other compounds of type **4**) at low temperature. Nevertheless, this behavior is not surprising owing to the, at best, moderate strength of the π-arene interaction. The same has been observed in previous studies on compounds of the group 14 series bearing terphenyl and related stabilizing ligands.[10]

3. X-Ray Structural Characterization of new compounds.

Crystallographic details. Low-temperature diffraction data were collected either on a Bruker D8 Quest APEX-III single crystal diffractometer with a Photon III detector and a IµS 3.0 microfocus X-ray source (**4-NTf$_2$·GaCl$_3$**, **4-GaCl$_4$**, **5** and **6**) or on a Bruker APEX-II CCD diffractometer (**3** and **4-NTf$_2$**) at the Instituto de Investigaciones Químicas, Sevilla. In both cases data were collected by means of ω and φ scans using monochromatic radiation λ(Mo Kα1) = 0.71073 Å. The diffraction images collected were processed and scaled using APEX-III or APEX-II software. The structures were solved with SHELXT and was refined against F^2 on all data by full-matrix least squares with SHELXL.[11] All non-hydrogen atoms were refined anisotropically. Hydrogen atoms were included in the model at geometrically calculated positions and refined using a riding model, unless otherwise noted. The isotropic displacement parameters of all hydrogen atoms were fixed to 1.2 times the U value of the atoms to which they are linked (1.5 times for methyl groups). In structure **3** the $GeCl_2$ and PMe_2 fragments were refined according to positional disordered across the P-Au-Ge axis with 1:1 occupancy. The corresponding Ge-Cl and P-C bond distances were refined to be similar by applying SADI instructions. The unit cell of **4-NTf$_2$** contains three independent benzene molecules, as well as the triflimide counteranion. Similarity restrains were applied to their anisotropic displacement parameters when required. There are also two independent benzene molecules in **4-GaCl$_4$**. C-C bond distances in one of those were restrained to a fix value (1.395Å), while their positions constrained as an ideal hexagon using AFIX instruction. The unit cell in structure **5** contains eight pentane molecules which were treated as a diffuse contribution to the overall scattering without specific atom positions by SQUEEZE/PLATON. The full numbering scheme of all the reported structures can be found in the full details of the X-ray structure determination (CIF), which are included as Supporting Information and have been deposited in the Cambridge Crystallographic Data Centre with no. 2013214-2013219. These data can be obtained free of charge from The Cambridge Crystallographic Data Centre via www.ccdc.cam.ac.uk/data_request/cif.

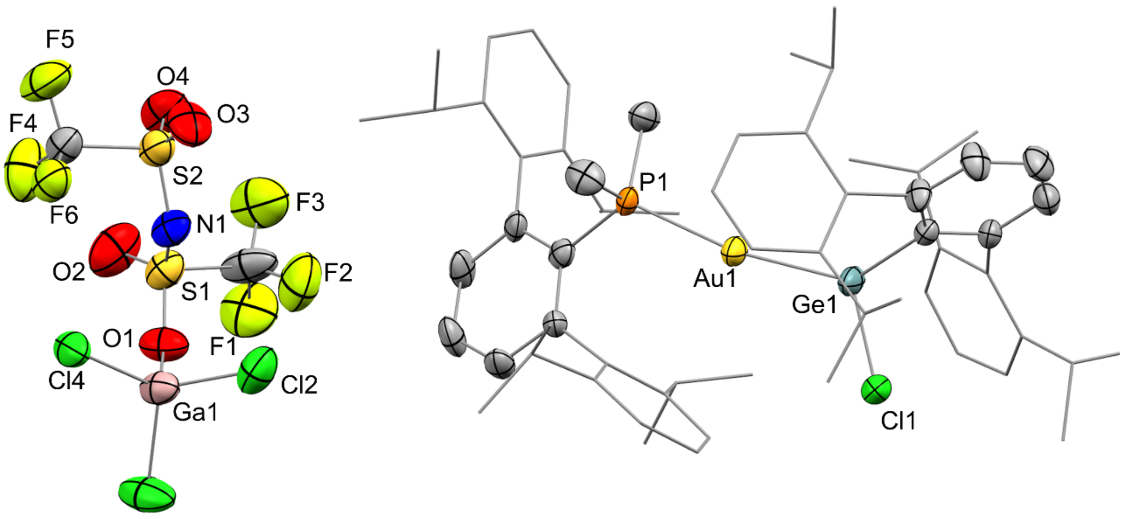

4-NTf$_2$GaCl$_3$

Figure S2. ORTEP diagram of compound **4-NTf$_2$·GaCl$_3$** where the NTf$_2$·GaCl$_3$ counteranion is shown. For the sake of clarity hydrogen atoms are excluded and some groups have been represented in wireframe format. Thermal ellipsoids are set at 50 % probability.

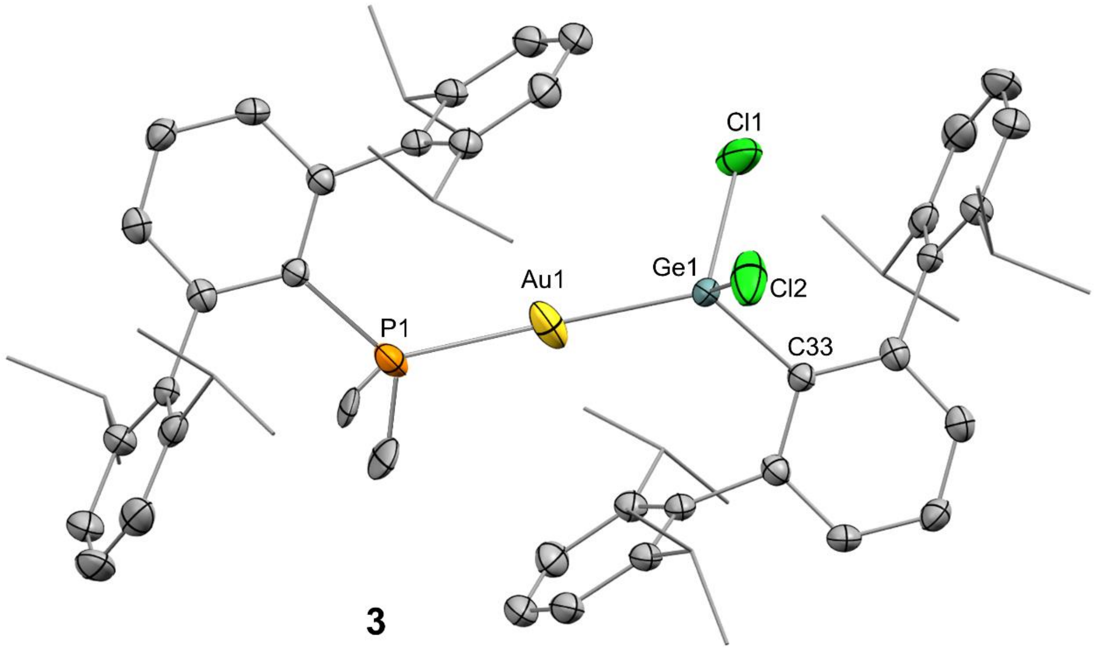

3

Figure S3. ORTEP diagram of compound **3**; for the sake of clarity hydrogen atoms and a dichloromethane solvent molecule are excluded and some groups have been represented in wireframe format. Thermal ellipsoids are set at 50 % probability.

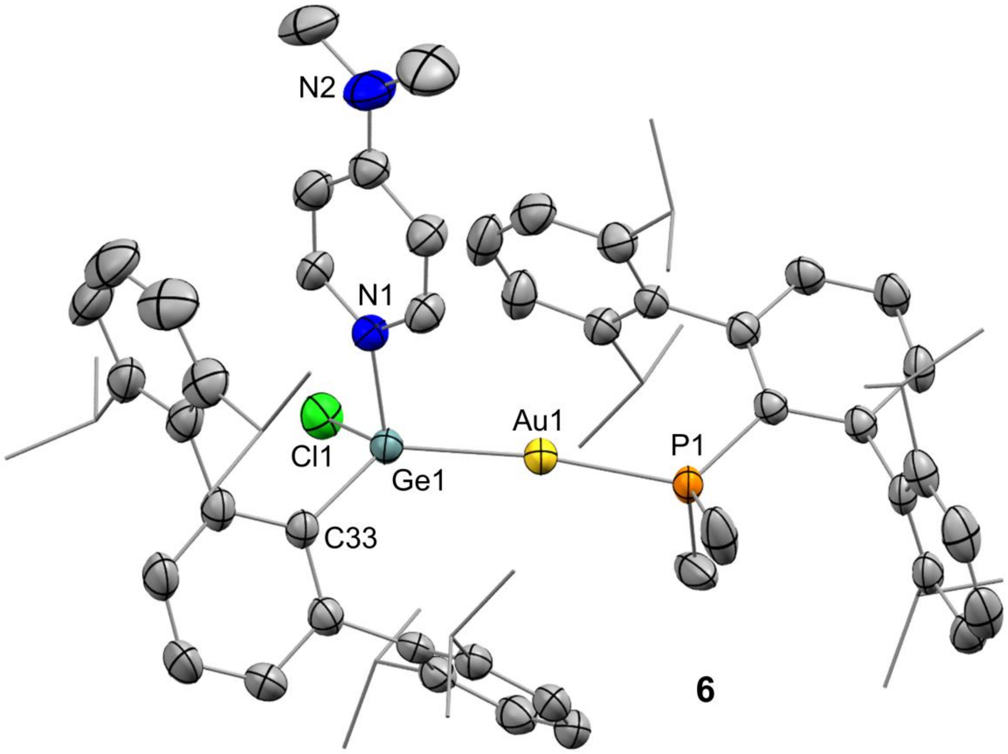

Figure S4. ORTEP diagram of compound **6**; for the sake of clarity hydrogen atoms, benzene solvent molecules and a triflimide counteranion are excluded, while some fragments have been represented in wireframe format. Thermal ellipsoids are set at 50 % probability.

Table S2. Crystal data and structure refinement for compounds **4-NTf$_2$**, **4-NTf$_2$·GaCl$_3$** and **4-GaCl$_4$**.

	**4·NTf$_2$**	**4-NTf$_2$·GaCl$_3$**	**4·GaCl$_4$**
formula	$C_{79}H_{95}AuClF_6GeNO_4PS_2$	$C_{67}H_{83}AuCl_4F_6GaGeNO_4PS_2$	$C_{71}H_{89}AuCl_5GaGeP$
fw	1636.65	1656.51	1489.91
cryst.size, mm	0.18 × 0.17 × 0.12	0.12 × 0.08 × 0.06	0.20 × 0.12 × 0.08
crystal system	Triclinic	Triclinic	Triclinic
space group	*P-1*	*P-1*	*P-1*
a, Å	13.1084 (7)	13.872 (3)	13.9555 (12)
b, Å	14.8663 (7)	15.281 (4)	14.8529 (15)
c, Å	20.9117 (10)	18.631 (4)	19.226 (2)
α, deg	87.729 (3)	92.562 (11)	87.943 (5)
β, deg	86.793 (3)	90.709 (10)	71.588 (4)
γ, deg	71.016 (2)	111.176 (9)	67.621 (3)
V, Å^3	3846.5 (3)	3677.5 (15)	3480.3 (6)
T, K	193	193	193
Z	2	2	2
ρ_{calc}, g cm^{-3}	1.413	1.496	1.422
μ, mm^{-1} (MoKα)	2.47	3.04	3.17
F(000)	1674	1670	1514
absorption corrections	multi-scan, 0.67-0.75	multi-scan, 0.37-0.49	multi-scan, 0.33-0.49
θ range, deg	2.7 – 25.9	1.9 – 25.0	2.0 – 25.2
no. of rflns measd	60266	91859	136691
R_{int}	0.035	0.157	0.084
no. of rflns unique	14632	8638	10654
no. of params / restraints	859 / 51	796 / 0	726 / 14
R_1 ($I > 2\sigma(I)$) [a]	0.031	0.067	0.053
R_1 (all data)	0.044	0.114	0.064
wR_2 ($I > 2\sigma(I)$)	0.067	0.140	0.136
wR_2 (all data)	0.072	0.167	0.145
Diff.Fourier.peaks min/max, eÅ^{-3}	-0.65 / 1.12	-1.77 / 1.98	-3.41 / 3.33
CCDC number	2013214	2013219	2013217

Table S3. Crystal data and structure refinement for compounds **3**, **5** and **6**.

	**3**	**5**	**6**
formula	$C_{64}H_{84}AuCl_6GeP$	$C_{64}H_{80}AgAuCl_2F_6GeNO_4PS_2$	$C_{83}H_{102}AuClF_6GeN_3O_4PS_2$
fw	1366.53	1584.70	1719.77
cryst.size, mm	0.16 × 0.14 × 0.12	0.20 × 0.16 × 0.12	0.19 × 0.19 × 0.10
crystal system	Monoclinic	Monoclinic	Triclinic
space group	$P2_1/c$	*Cc*	*P-1*
a, Å	12.3080 (6)	12.6243 (4)	12.7244 (18)
b, Å	10.7012 (5)	24.9004 (9)	16.935 (3)
c, Å	24.1596 (11)	24.4163 (8)	20.354 (3)
α, deg	90	90	81.502 (6)
β, deg	99.341 (2)	97.325 (1)	72.855 (6)
γ, deg	90	90	76.579 (6)
V, Å^3	3139.9 (3)	7612.6 (4)	4061.6 (11)
T, K	193	193	193
Z	2	4	2
ρ_{calc}, g cm^{-3}	1.445	1.383	1.406
μ, mm^{-1} (MoKα)	3.13	2.77	2.34
F(000)	1392	3184	1764
absorption corrections	multi-scan, 0.66-0.75	multi-scan, 0.62-0.75	multi-scan, 0.54-0.75
θ range, deg	2.6 – 30.6	1.9 – 26.0	2.1 – 26.4
no. of rflns measd	61110	72327	131611
R_{int}	0.041	0.051	0.070
no. of rflns unique	9582	14132	16607
no. of params / restraints	366 / 2	767 / 11	915 / 12
R_1 ($I > 2\sigma(I)$) [a]	0.043	0.026	0.038
R_1 (all data)	0.076	0.029	0.054
wR_2 ($I > 2\sigma(I)$)	0.104	0.061	0.114
wR_2 (all data)	0.118	0.063	0.131
Diff.Fourier.peaks min/max, eÅ^{-3}	-3.30 / 0.74	-0.61 / 0.81	-1.24 / 1.16
CCDC number	2013216	2013215	2013218

Main packing interactions involving counteranions in the structures of 4-NTf$_2$, 4-NTf$_2$·GaCl$_3$ and 4-GaCl$_4$.

A range of weak interactions in the solid state and dominated by the three investigated counteranions is most likely responsible for the different conformations observed for cation **4**$^+$. Figure S5 collects those interactions and their defining geometric parameters. In **4·NTf$_2$** there are two C-H···O interactions for each triflimidate anion involving C(sp^2)-H termini of terphenyl rings of two different **4**$^+$ cations. One of the latter additionally participates in a C-H···F contact with the same triflimidate anion which connects by the same type of interaction a third cation **4**$^+$. The structure of **4·NTf$_2$·GaCl$_3$** involves pairing of the uncommon [NTf$_2$·GaCl$_3$]$^-$ anion to a single **4**$^+$ cation through two C-H···O contacts, as well as a C-F···F-C interaction with an adjacent anion. Finally, the structure of **4·GaCl$_4$** is characterized by three C-H···Cl interactions between two of the chlorine atoms of GaCl$_4^-$ with three **4**$^+$ cations. All C-H···X and F···F interactions exhibit normal interatomic distances and angles.[12]

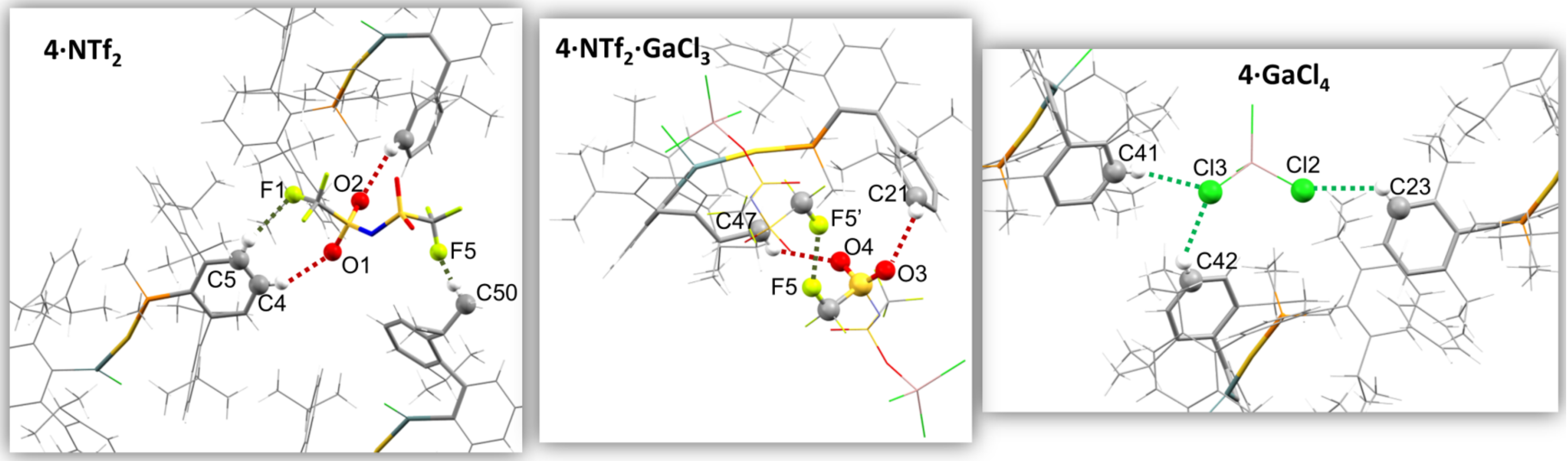

Type	$d_{C\text{-}X}$ (Å)	$d_{X\cdots H}$ (Å)	C—H···X (°)
C-H···F	3.556	2.667	156.0
C-H···F	3.413	2.638	136.2
C-H···O	3.389	2.510	153.9
C-H···O	3.326	2.537	140.6

Type	$d_{C\text{-}X}$ (Å)	$d_{X\cdots H}$ (Å)	C—H···X (°)
C-H···O	3.354	2.602	133.6
C-H···O	3.407	2.654	136.5

Type	$d_{F\text{-}F}$ (Å)
F···F	2.871

Type	$d_{C\text{-}X}$ (Å)	$d_{X\cdots H}$ (Å)	C—H···X (°)
C-H···Cl	3.758	2.859	158.3
C-H···Cl	3.515	2.893	124.1
C-H···Cl	3.685	2.900	148.7

Figure S5. Representation of main solid state weak interactions in structures **4-NTf$_2$**, **4-NTf$_2$·GaCl$_3$** and **4-GaCl$_4$**, highlighting in sphere format the atoms directly involved in the contacts.

4. Computational details

Geometry optimization of minima and transition states was carried out with the Gaussian software package.[13] Optimizations were carried out without symmetry restrictions using DFT methods. The PBE0 functional[14] was used with empirical dispersion taken into account by adding the D3 version of Grimme's dispersion with Becke-Johnson damping.[15] The 6-31g(d,p) basis set[16] was used for non-metal atoms and the Au atoms were described with the SDD basis and associated electron core potential (ECP).[17] Bulk solvent effects (benzene) were included during optimization with the SMD continuum model.[18] The extended wavefunction .wfx and NBO .47 files were calculated on previously optimized geometries but using the triple-ζ basis set def2-TZVP[19] basis for all atoms, which includes and ECP for Au.[20] Wavefunction analysis and NBO analysis were performed with the Multifwn code[21] and the NBO6.0[22] software respectively. The CYLview visualization software has been used to create some of the figures.[23]

The X-Ray structure of the cation of **4-NTf$_2$** was optimized freely in bulk benzene resulting in a geometry, **4s**$^+$, with a shortest Ge···C_{aryl} distance of 2.40Å, which is 0.09 Å shorter than the experimental result. Inclusion of one explicit triflimidate anion in the calculation yields the expected ion pair and its formation is exergonic by 17.1 kcal·mol^{-1} (with respect to the ions at infinite distance in bulk benzene solution). Also, this interaction further shortened the Ge···C_{aryl} distance to 2.32 Å, evincing the influence of the counteranion in the geometry of the cation. Furthermore, N-coordination of this anion to the Ge atom is exergonic by 1.02 kcal·mol^{-1} with respect to the ion pair in agreement with the diffusion NMR experiments.

When the X-Ray structures of **4-NTf$_2$·GaCl$_3$** and **4-GaCl$_4$** were used as starting points, free optimizations of the cations, the shortest Ge···C_{aryl} distances collapsed to values significantly shorter than the experimental. Indeed, fully relaxed Potential Energy Surface scans along Ge···C_{aryl} distance coordinates revealed one absolute minimum at shortest Ge···C_{aryl} distances close to that found in **4-NTf$_2$** and no local minima for scenarios with longer Ge···C_{aryl} distances.

Figure S5 shows the results of two of these scans. The blue dots are energies for geometries starting from that of **4s**$^+$, as a function of the Ge···C_{aryl} distance. The orange dots correspond to results obtained starting from a geometry with longer Ge···C_{aryl} distances. One striking result from these PES scans is that the shortest Ge···C_{aryl} distance at the absolute minimum is dependant of the initial geometry used in the calculation, which hints at subtle equilibria between attractive and repulsive interactions within the cation. In addition, geometries with longer Ge···C_{aryl} distances must be stabilized by intramolecular interactions, not considered in the present study.

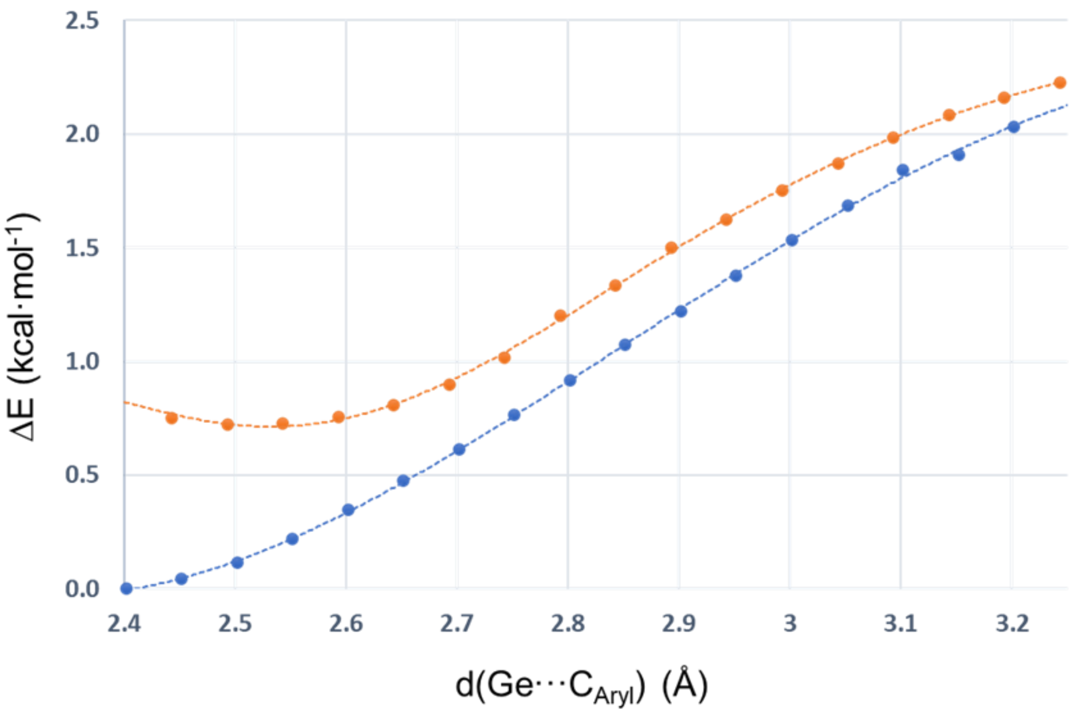

Figure S6. Fully relaxed Potential Energy Scan along Ge···C_{aryl} distance coordinates.

Since the calculations could not account for geometries with intermediate and long Ge···C_{aryl} distances when no geometric constrains were imposed, new optimizations starting from the X-ray structures of the cations of **4-NTf$_2$·GaCl$_3$** and **4-GaCl$_4$** were performed, but this time the shortest Ge···C_{aryl} distance was fixed to the experimental value in each case to yield cations **4i**$^+$ and **4l**$^+$. The resulting geometries, together with that of **4s**$^+$, were used in subsequent localized orbital (NBO) and electron density (AIM) analysis. Selected data are shown in Tables S4-S6.

Table S4. NPA charges and Wiberg Bond Indices.

		**4s**$^+$	**4i**$^+$	**4l**$^+$
**NPA charges (e$^-$)**				
**fragments**	PAu$^+$	0.633	0.644	0.643
	ArGeCl	0.367	0.356	0.357
**WBO**				
**bond / interaction**	Au—Ge	0.553	0.557	0.560
	Ge···C_{aryl}	0.224	0.124	0.073

Table S5. Relevant NBO results including major donor-acceptor interactions.

NBO analysis	Donor NBO / occupancy (e)	Acceptor NBO / occupancy	ΔE_{ij} kcal·mol^{-1}	NLMO
**4s$^+$**	LP_{Ge}(4s) / 1.50	LV (s) Au / 0.81	350.5	23.64 % Au
	$\pi_{C=C}$ / 1.50	LV (4p) Ge / 0.33	44.6	8.27 % Ge
**4i$^+$**	LP_{Ge}(4s) / 1.62	$\sigma^*_{Au—P}$ / 0.42	223.8	14.09 % Au
	$\pi_{C=C}$ / 1.61	LV (4p) Ge / 0.30	17.87	4.17 % Ge
**4l$^+$**	LP_{Ge}(4s) / 1.62	$\sigma^*_{Au—P}$ / 0.46	220.0	14.14 % Au
	$\pi_{C=C}$ / 1.62	LV (4p) Ge / 0.29	9.59	2.06 % Ge

Table S6. AIM indicators at relevant bcps. Electron density ρ_b (e·bohr^{-3}); ellipticity ε_b.

	**bond /interaction**	ρ_b	ε_b
**4s$^+$**	Au—Ge	0.0922	0.0138
	Ge···C_{aryl}	0.0489	0.2744
**4i$^+$**	Au—Ge	0.0925	0.0096
	Ge···C_{aryl}	0.0255	1.2729
**4l$^+$**	Au—Ge	0.0919	0.0155
	Ge···C_{aryl}	-	-

Figure S6 reveals Natural Localized Molecular Orbitals (NLMO) related to $\pi_{C=C}\rightarrow p_{Ge}$ donation in cations **4**$^+$. The extent of participation of the Ge atom in each case is evident from the shape of the orbitals. In addition, the LUMO of **4l**$^+$ (Figure S7) is an almost pure vacant p orbital localized on Ge, which is consistent with our description of the Ge cation of **4-GaCl$_4$** as containing a naked Ge cation, with no interaction with arene electron density.

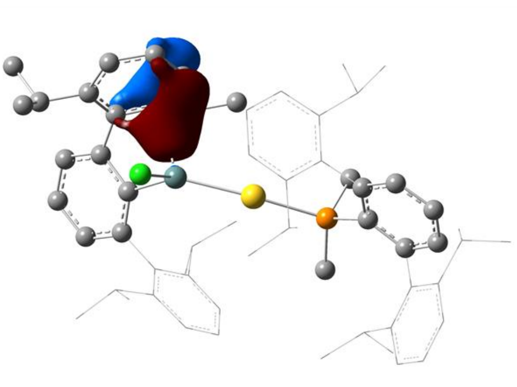

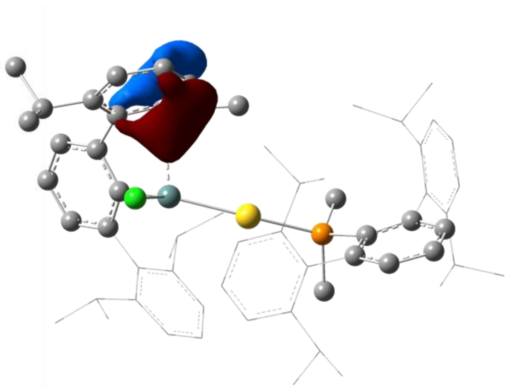

4s$^+$ **4i**$^+$

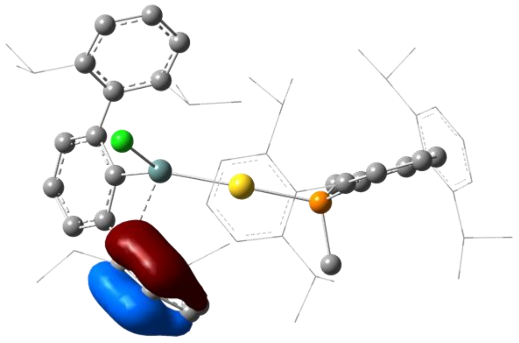

4l$^+$

Figure S7. NLMOs involved in $\pi_{C=C}\rightarrow p_{Ge}$ electron donation in cations **4**$^+$.

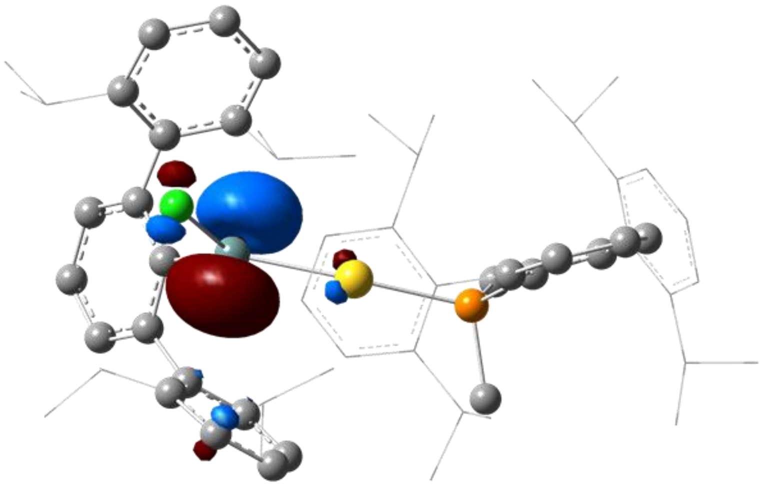

4l$^+$

Figure S8. LUMO of cation **4l**$^+$.

Figures S8-S10 show molecular graphs of the different cations including relevant bcps and plots of the same results on the corresponding Au—Ge—C_{aryl} planes overlaid on the Laplacian of their electron density. The plot corresponding to cation **4i**$^+$ (Figure S9) reveals one bcp and bond path connecting the expected Ge and C_{aryl} atoms. Interestingly, the ellipticity at the bcp is high at 1.27. The ellipticity at the bcp is defined as $\boldsymbol{\varepsilon = \lambda_1/\lambda_2-1}$**,** where λ_1 and λ_2 are the curvatures (or the eigenvalues of the Hessian) of the electron density perpendicular to the bond path at the bcp, $\boldsymbol{\rho_b}$. For cylindrical bonds, this curvature is expected to be cero, since $\lambda_1 = \lambda_2$. As can be seen in Figure S7, a ring critical point (in orange) is close to the Ge···C_{aryl} bond critical point of **4i**$^+$, causing the ellipticity to increase.[24] This situation is close to what has been termed a catastrophe scenario and indicates an unstable bond. The catastrophe scenario arises from the elongation of the Ge···C_{aryl} bond, which causes the bond critical point to migrate towards the ring critical point (of the ring formed by the Ge atom and four atoms or the terphenyl moiety including the Ge···C_{aryl} carbon). At the point of coalescence of both critical points, the "bifurcation catastrophe" is the frontier between two molecular structures, one with a Ge···C_{aryl} interaction and another one with a "naked" Ge cation.

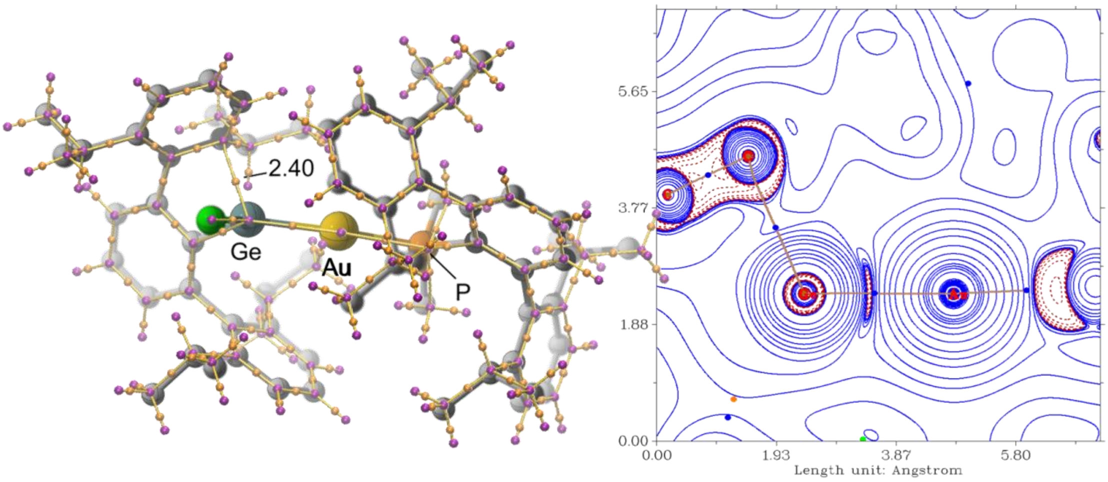

Figure S9. DFT-optimized geometry of **4s**$^+$ overlaid by its AIM-molecular graph, (3,-1) bcps with $\rho < 0.02$ a.u. and related bond paths are omitted (left) and representative bcps and bond paths on the electron density of calculated **4s**$^+$, overlaid on the laplacian on the electron density, $\nabla_2\boldsymbol{\rho}$, in the Au1-Ge1-C40 plane.

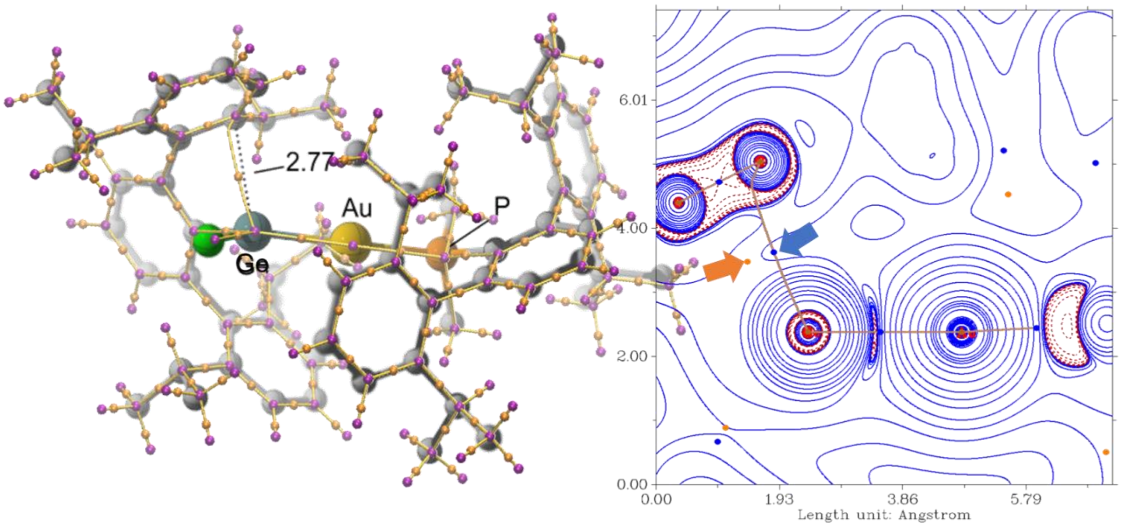

Figure S10. DFT-optimized geometry of **4i**$^+$ overlaid by its AIM-molecular graph, (3,-1) bcps with ρ < 0.02 a.u. and related bond paths are omitted (left) and representative bcps and bond paths on the electron density of calculated **4i**$^+$, overlaid on the laplacian on the electron density, $\nabla_2\boldsymbol{\rho}$, in the Au1-Ge1-C40 plane.

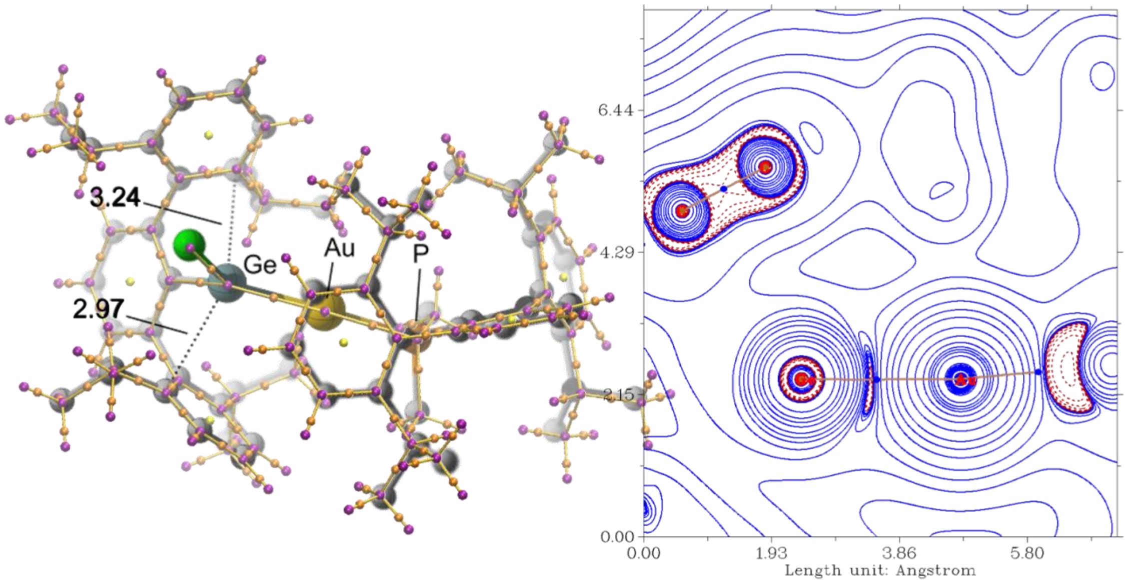

Figure S11. DFT-optimized geometry of **4l**$^+$**b** overlaid by its AIM-molecular graph, (3,-1) bcps with ρ < 0.02 a.u. and related bond paths are omitted (left) and representative bcps and bond paths on the electron density of calculated **4l**$^+$**b**, overlaid on the laplacian on the electron density, $\nabla_2\boldsymbol{\rho}$, in the Au1-Ge1-C40 plane. Notice that in this case the Ge···Caryl distance fixed in the calculation corresponds to the same atoms as in **4s**$^+$ and **4i**$^+$, and not to the shortest Ge···Caryl distance found in the X-Ray analysis of **4-GaCl$_4$**.

5. NMR spectra of new compounds

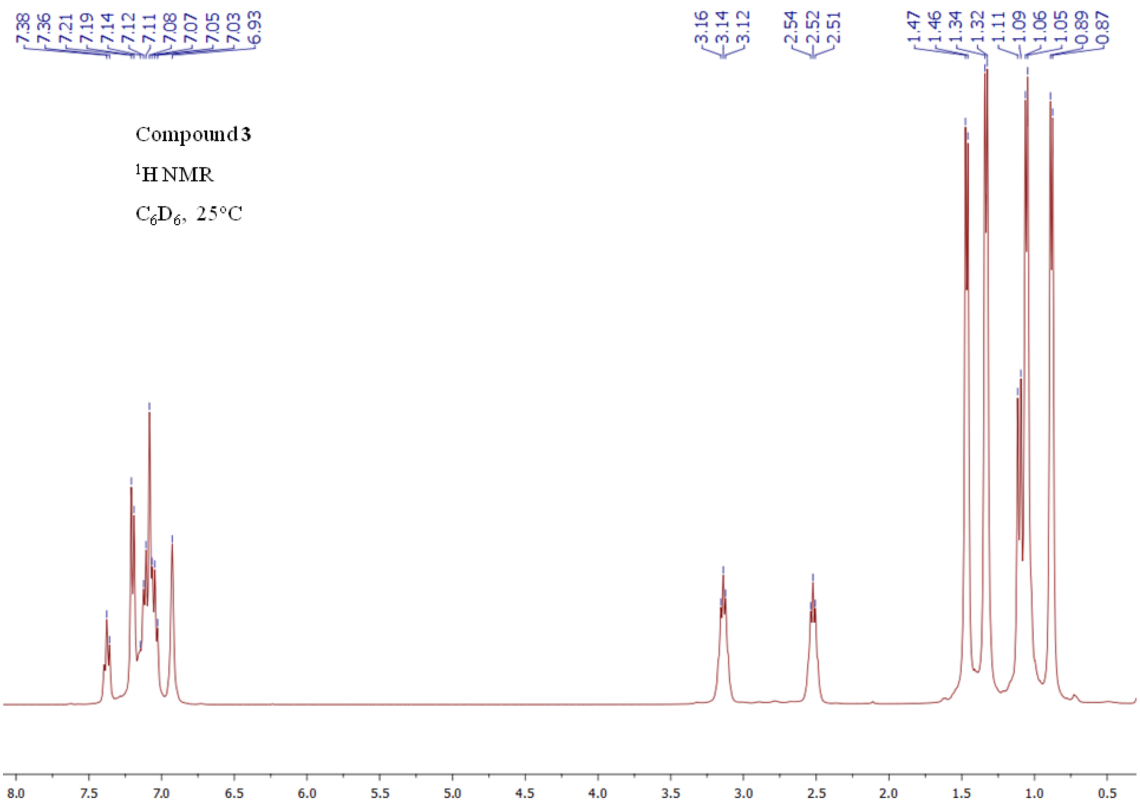

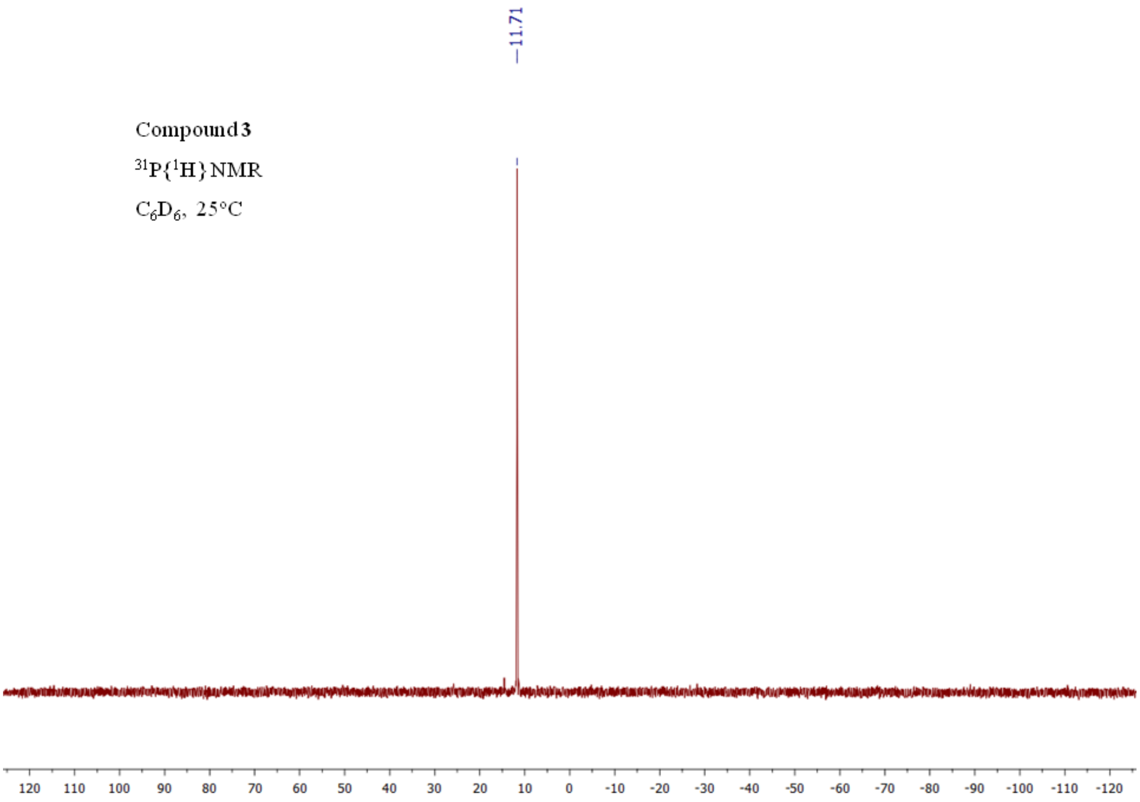

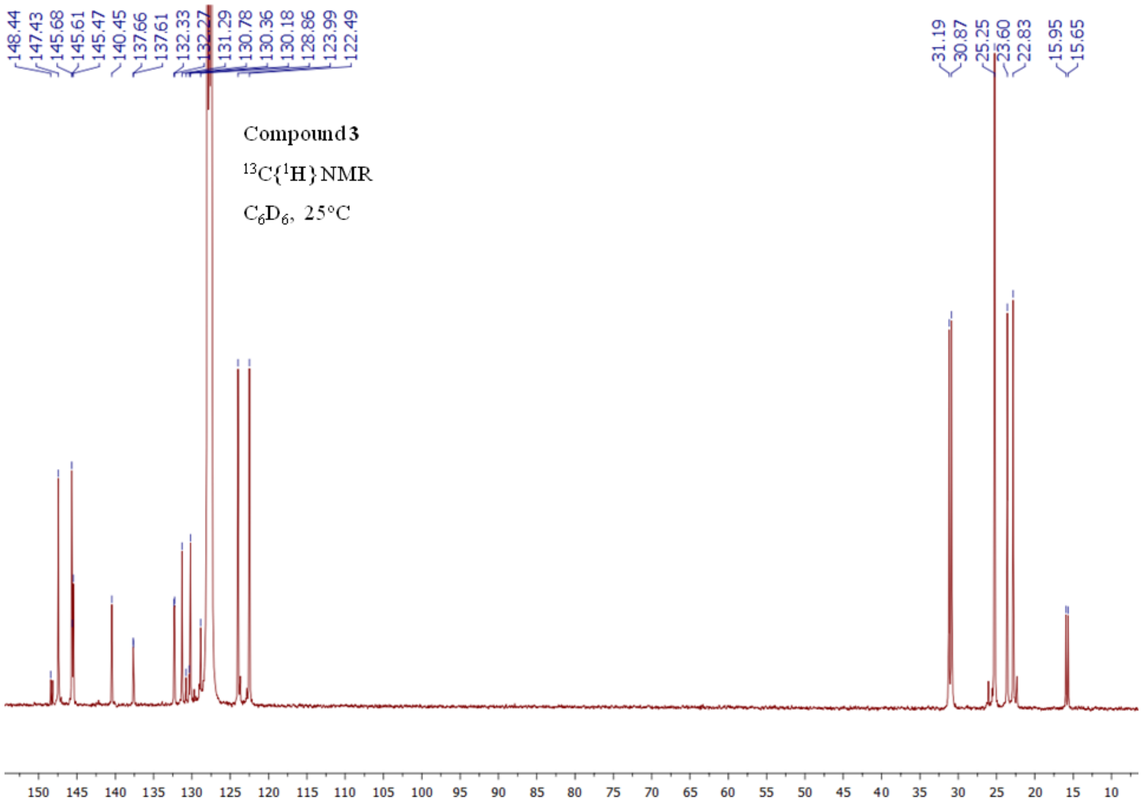
148.44
147.43
145.68
145.61
145.47
140.45
137.66
137.61
132.33
132.27
131.29
130.78
130.36
130.18
128.86
123.99
122.49
31.19
30.87
25.25
23.60
22.83
15.95
15.65
Compound 3
13C{1H} NMR
C6D6, 25°C
150 145 140 135 130 125 120 115 110 105 100 95 90 85 80 75 70 65 60 55 50 45 40 35 30 25 20 15 10

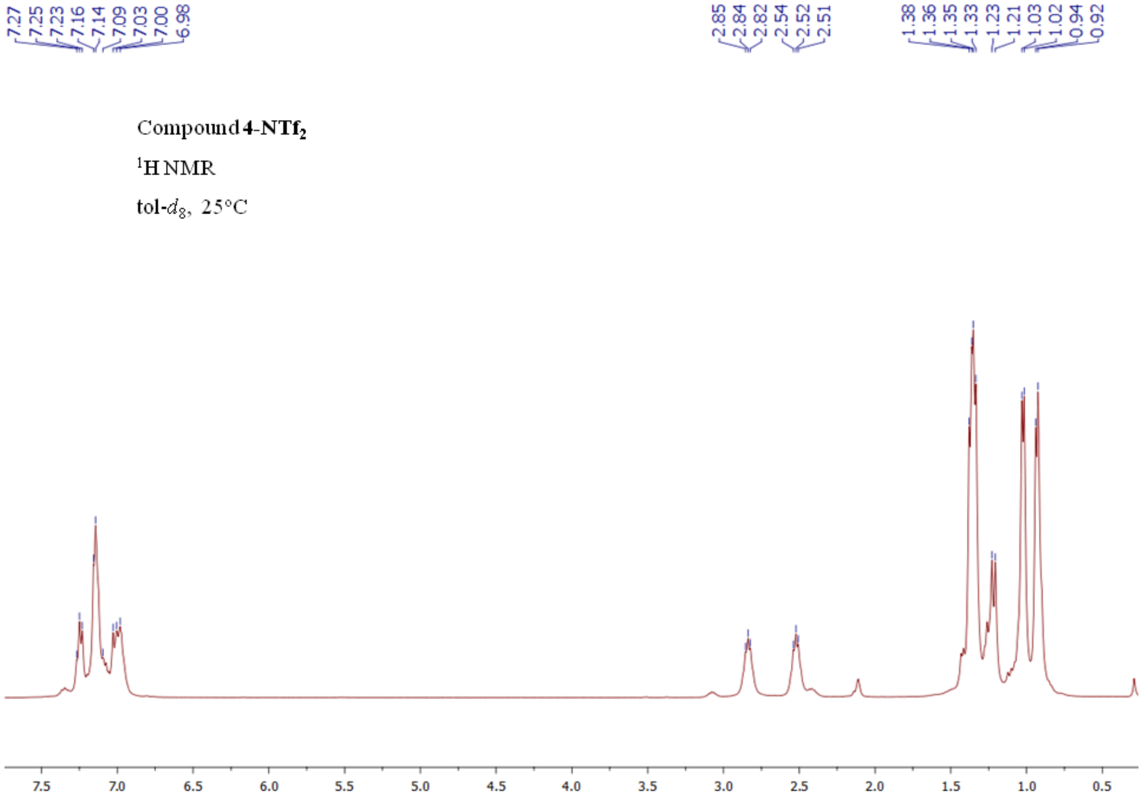
7.27
7.25
7.23
7.16
7.14
7.09
7.03
7.00
6.98
2.85
2.84
2.82
2.54
2.52
2.51
1.38
1.36
1.35
1.33
1.23
1.21
1.03
1.02
0.94
0.92
Compound 4-NTf2
1H NMR
tol-d8, 25°C
7.5 7.0 6.5 6.0 5.5 5.0 4.5 4.0 3.5 3.0 2.5 2.0 1.5 1.0 0.5

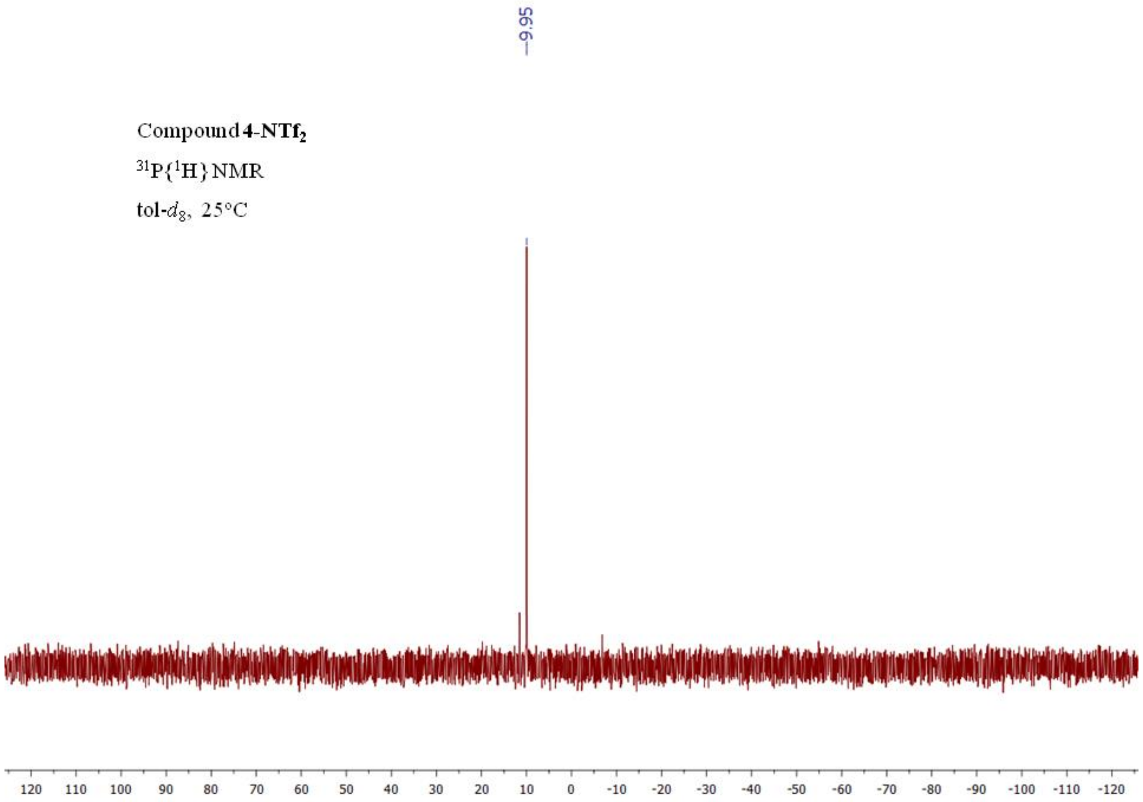
9.95
Compound 4-NTf2
31P{1H} NMR
tol-d8, 25°C

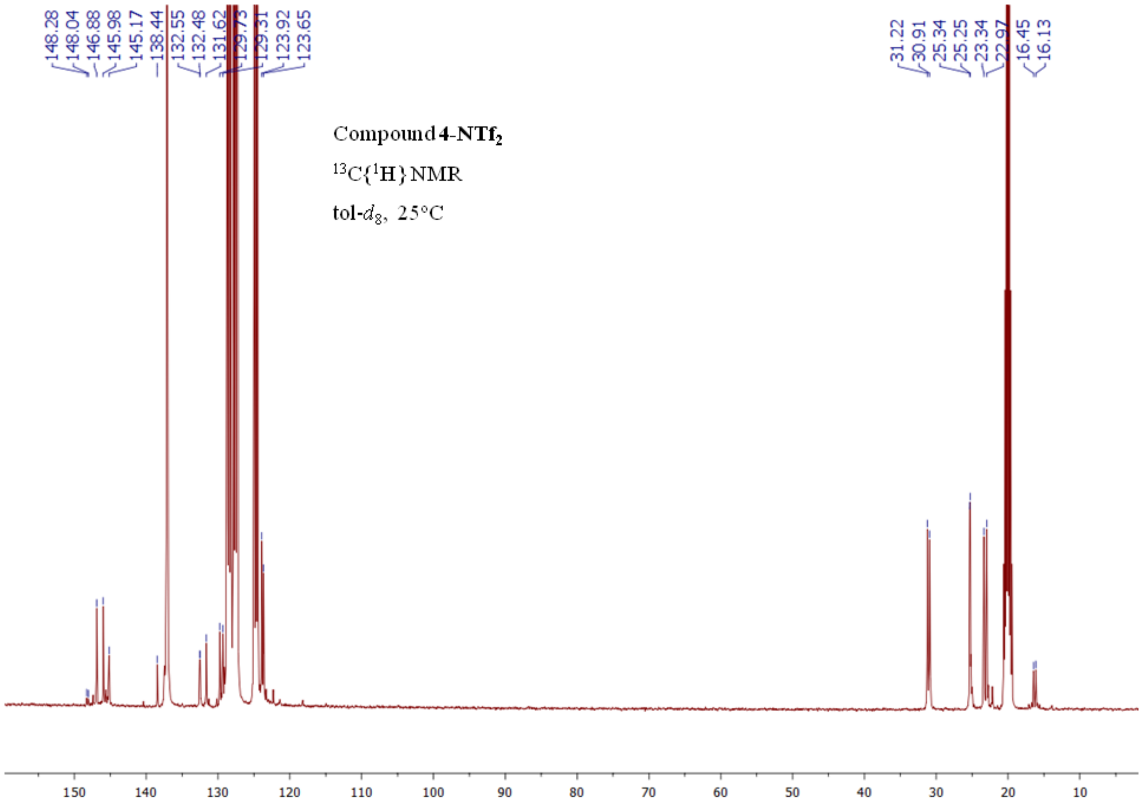
148.28
148.04
146.88
145.98
145.17
138.44
132.55
132.48
131.62
129.73
129.31
123.92
123.65
31.22
30.91
25.34
25.25
23.34
22.97
16.45
16.13
Compound 4-NTf2
13C{1H} NMR
tol-d8, 25°C

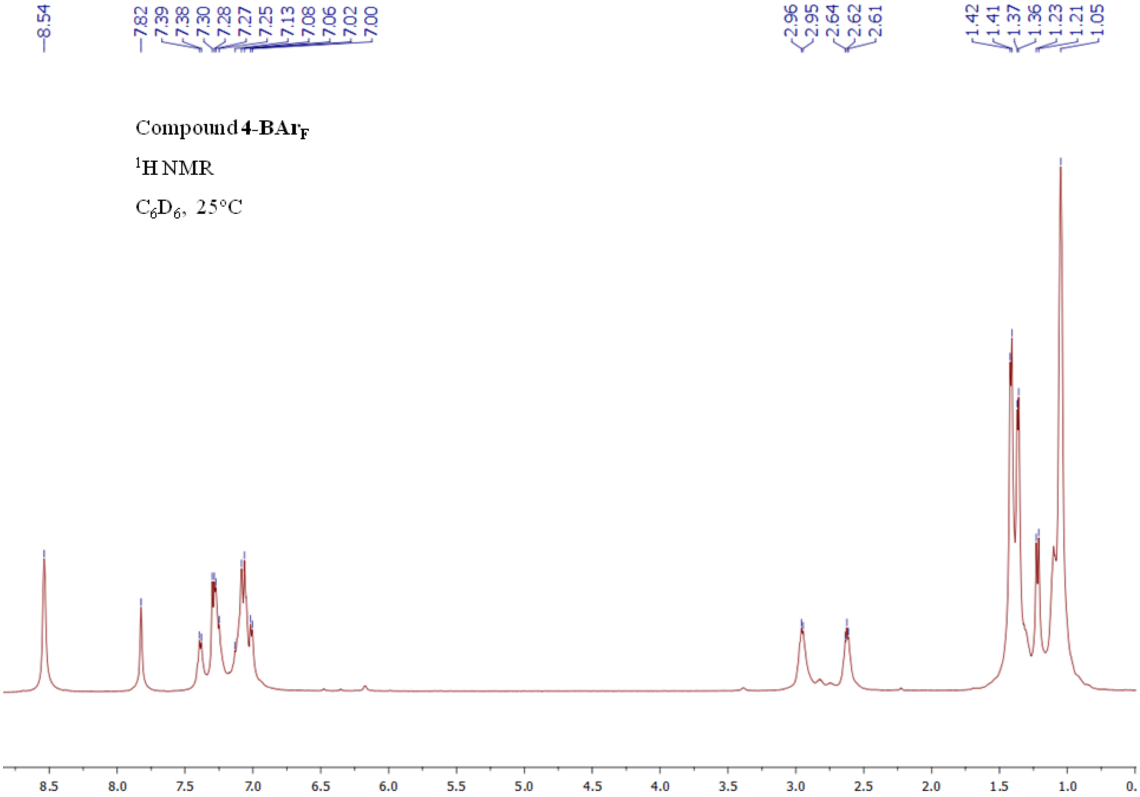
8.54
7.82
7.39
7.38
7.30
7.28
7.27
7.25
7.13
7.08
7.06
7.02
7.00
2.96
2.95
2.64
2.62
2.61
1.42
1.41
1.37
1.36
1.23
1.21
1.05
Compound **4-BAr$_F$**
^{1}H NMR
C_6D_6, 25°C
8.5 8.0 7.5 7.0 6.5 6.0 5.5 5.0 4.5 4.0 3.5 3.0 2.5 2.0 1.5 1.0 0.

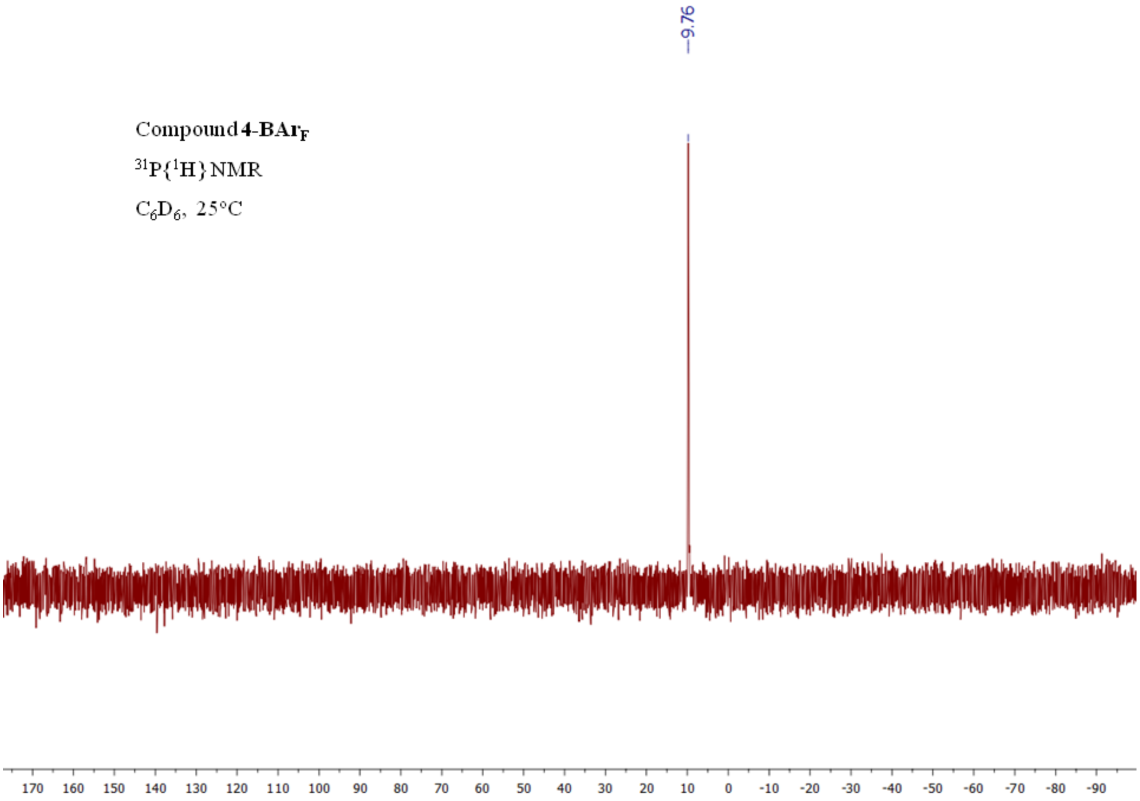
9.76
Compound **4-BAr$_F$**
^{31}P{^{1}H} NMR
C_6D_6, 25°C
170 160 150 140 130 120 110 100 90 80 70 60 50 40 30 20 10 0 -10 -20 -30 -40 -50 -60 -70 -80 -90

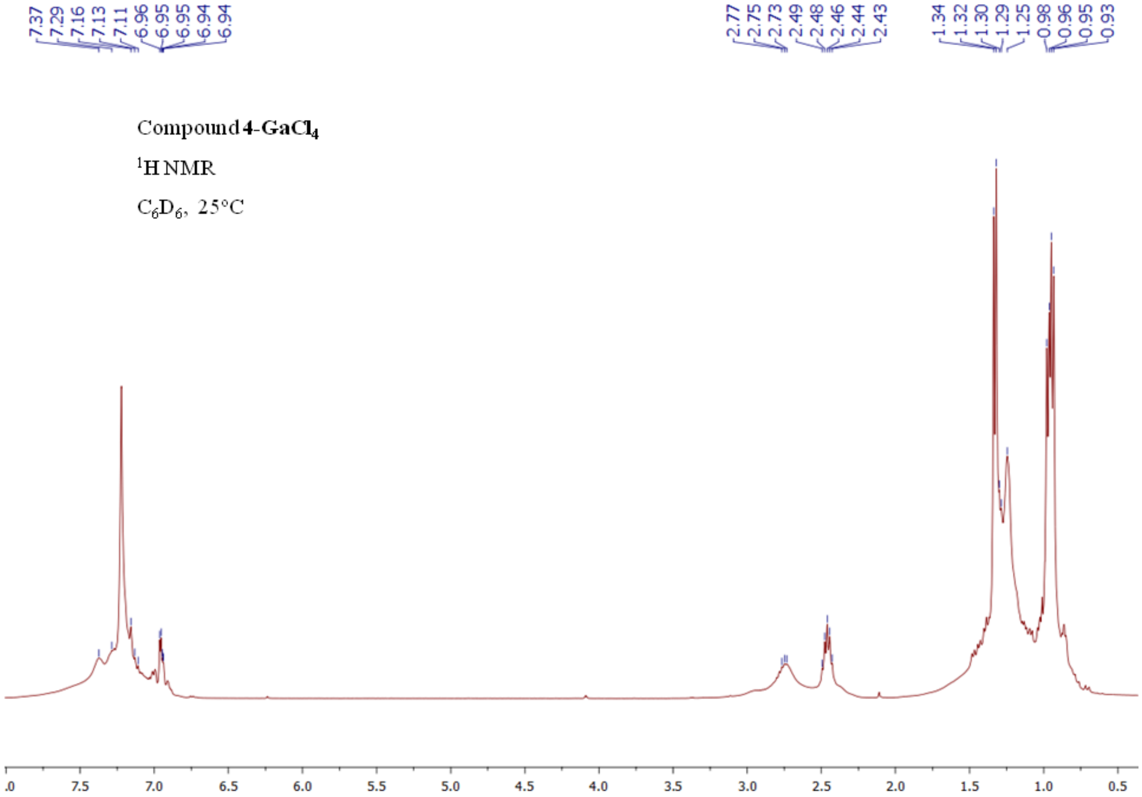
7.37
7.29
7.16
7.13
7.11
6.96
6.95
6.95
6.94
6.94
2.77
2.75
2.73
2.49
2.48
2.46
2.44
2.43
1.34
1.32
1.30
1.29
1.25
0.98
0.96
0.95
0.93
Compound 4-GaCl4
1H NMR
C6D6, 25°C
.0
7.5
7.0
6.5
6.0
5.5
5.0
4.5
4.0
3.5
3.0
2.5
2.0
1.5
1.0
0.5

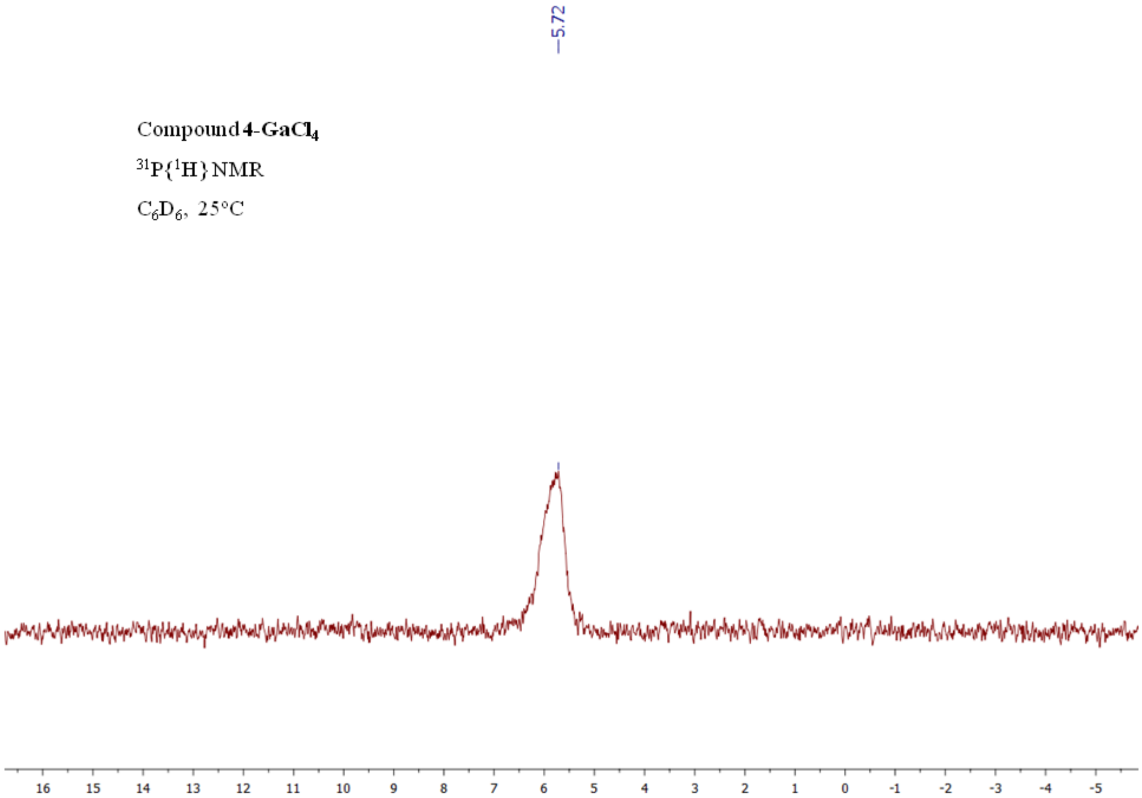
5.72
Compound 4-GaCl4
31P{1H} NMR
C6D6, 25°C
16
15
14
13
12
11
10
9
8
7
6
5
4
3
2
1
0
-1
-2
-3
-4
-5

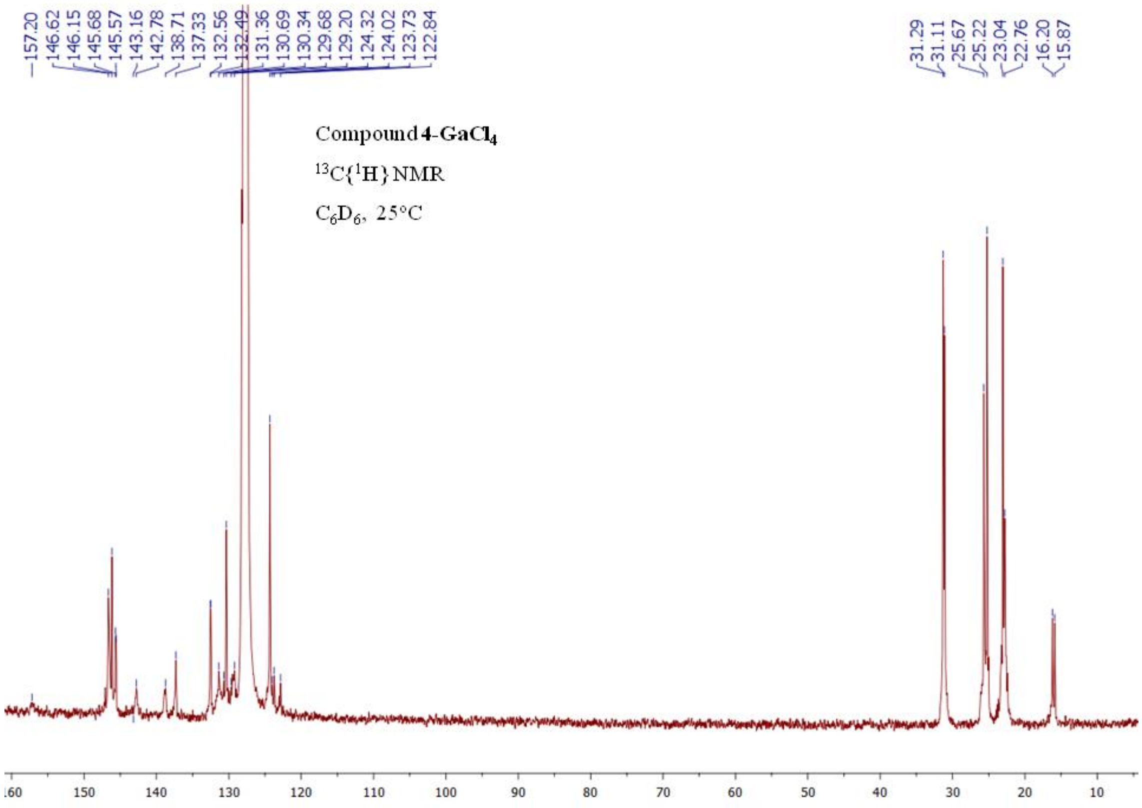
157.20
146.62
146.15
145.68
145.57
143.16
142.78
138.71
137.33
132.56
132.49
131.36
130.69
130.34
129.68
129.20
124.32
124.02
123.73
122.84
31.29
31.11
25.67
25.22
23.04
22.76
16.20
15.87
Compound 4-GaCl4
13C{1H} NMR
C6D6, 25°C
160 150 140 130 120 110 100 90 80 70 60 50 40 30 20 10

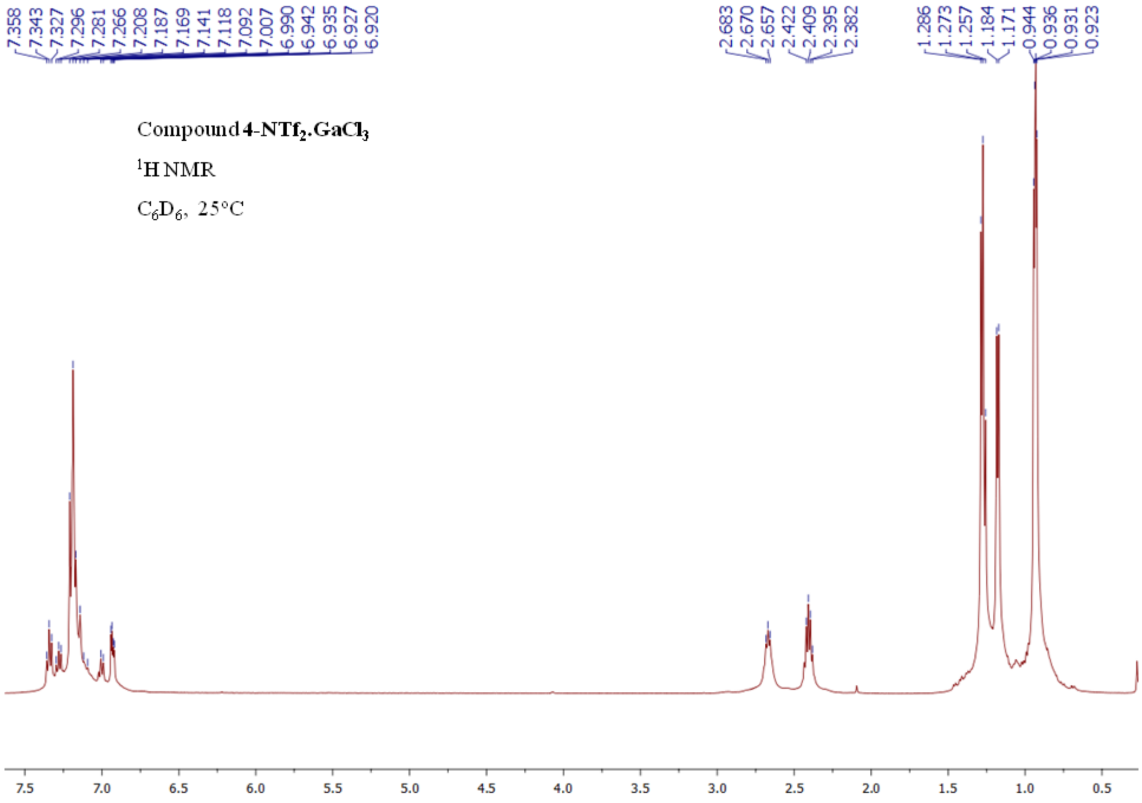
7.358
7.343
7.327
7.296
7.281
7.266
7.208
7.187
7.169
7.141
7.118
7.092
7.007
6.990
6.942
6.935
6.927
6.920
2.683
2.670
2.657
2.422
2.409
2.395
2.382
1.286
1.273
1.257
1.184
1.171
0.944
0.936
0.931
0.923
Compound 4-NTf2.GaCl3
1H NMR
C6D6, 25°C
7.5 7.0 6.5 6.0 5.5 5.0 4.5 4.0 3.5 3.0 2.5 2.0 1.5 1.0 0.5

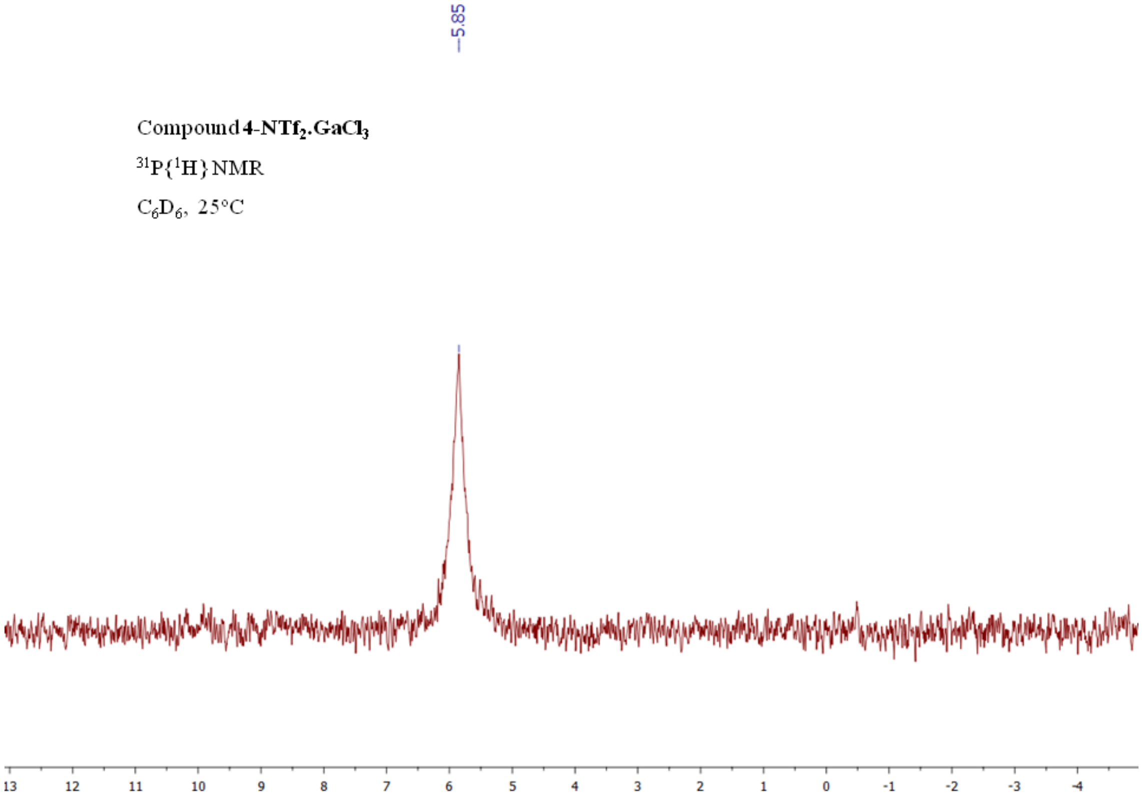
-5.85
Compound 4-NTf2.GaCl3
31P{1H} NMR
C6D6, 25°C
13
12
11
10
9
8
7
6
5
4
3
2
1
0
-1
-2
-3
-4

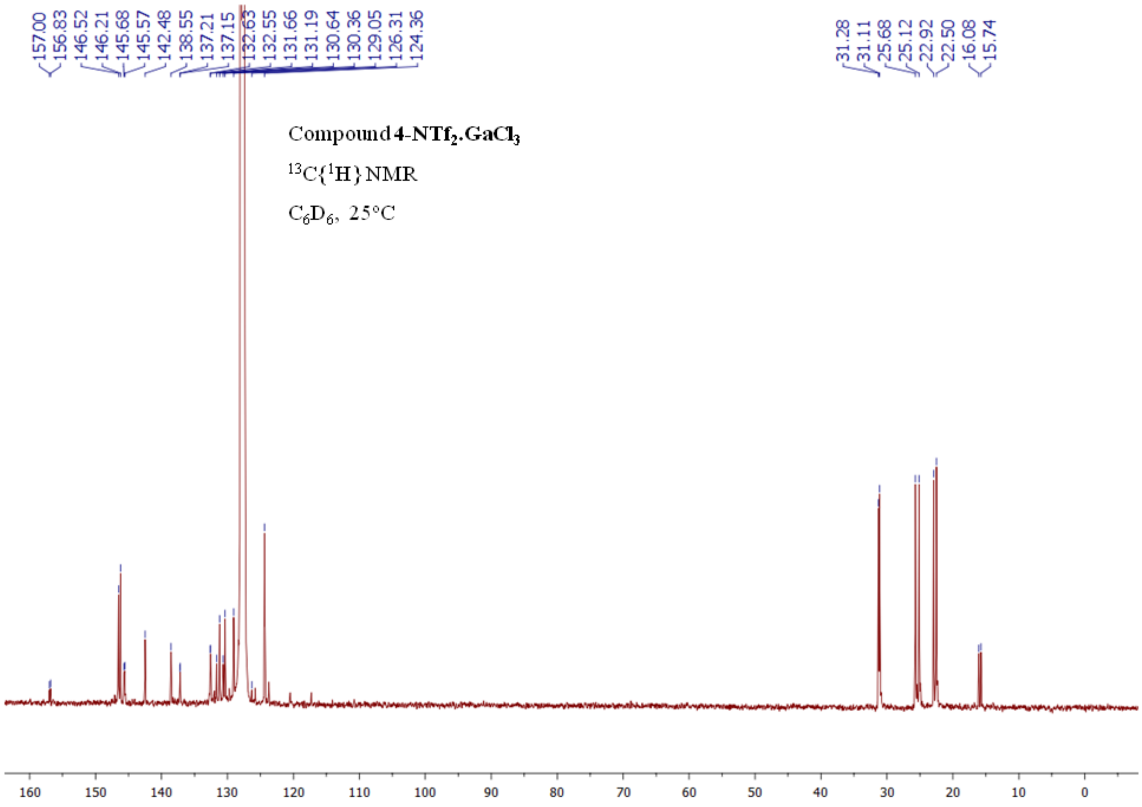
157.00
156.83
146.52
146.21
145.68
145.57
142.48
138.55
137.21
137.15
132.63
132.55
131.66
131.19
130.64
130.36
129.05
126.31
124.36
31.28
31.11
25.68
25.12
22.92
22.50
16.08
15.74
Compound 4-NTf2.GaCl3
13C{1H} NMR
C6D6, 25°C
160
150
140
130
120
110
100
90
80
70
60
50
40
30
20
10
0

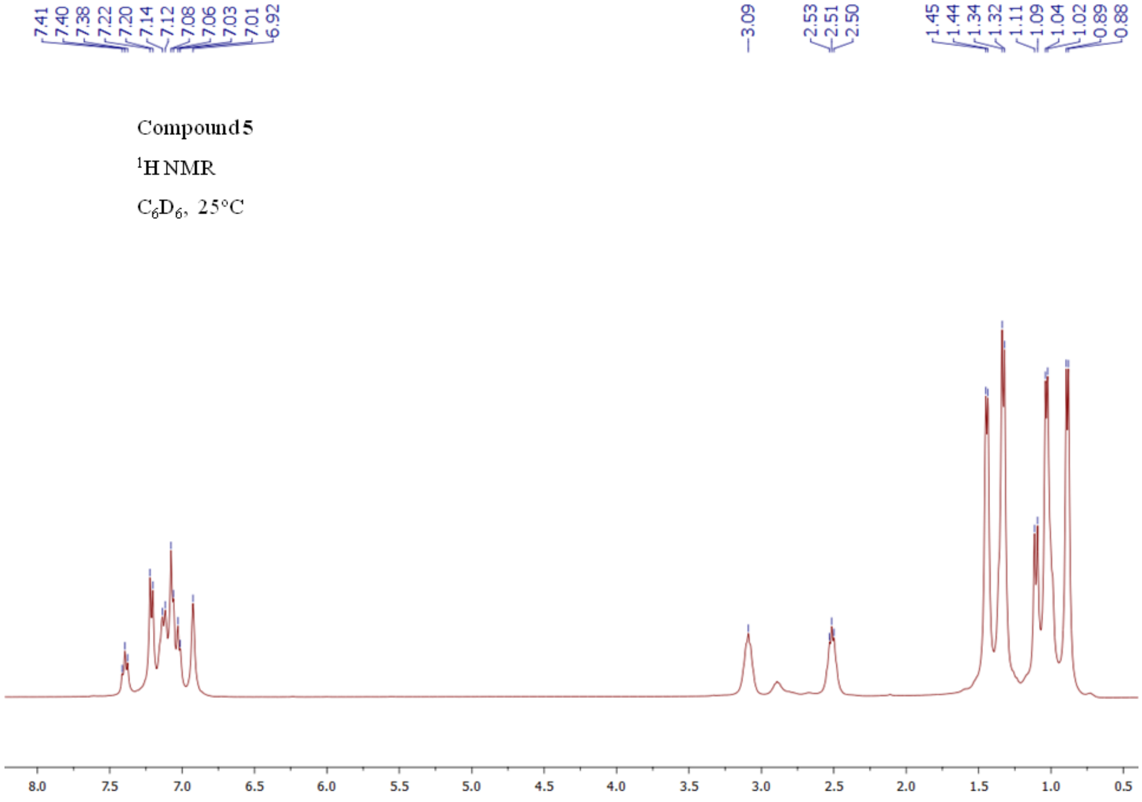
7.41
7.40
7.38
7.22
7.20
7.14
7.12
7.08
7.06
7.03
7.01
6.92
3.09
2.53
2.51
2.50
1.45
1.44
1.34
1.32
1.11
1.09
1.04
1.02
0.89
0.88
Compound 5
1H NMR
C6D6, 25°C
8.0 7.5 7.0 6.5 6.0 5.5 5.0 4.5 4.0 3.5 3.0 2.5 2.0 1.5 1.0 0.5

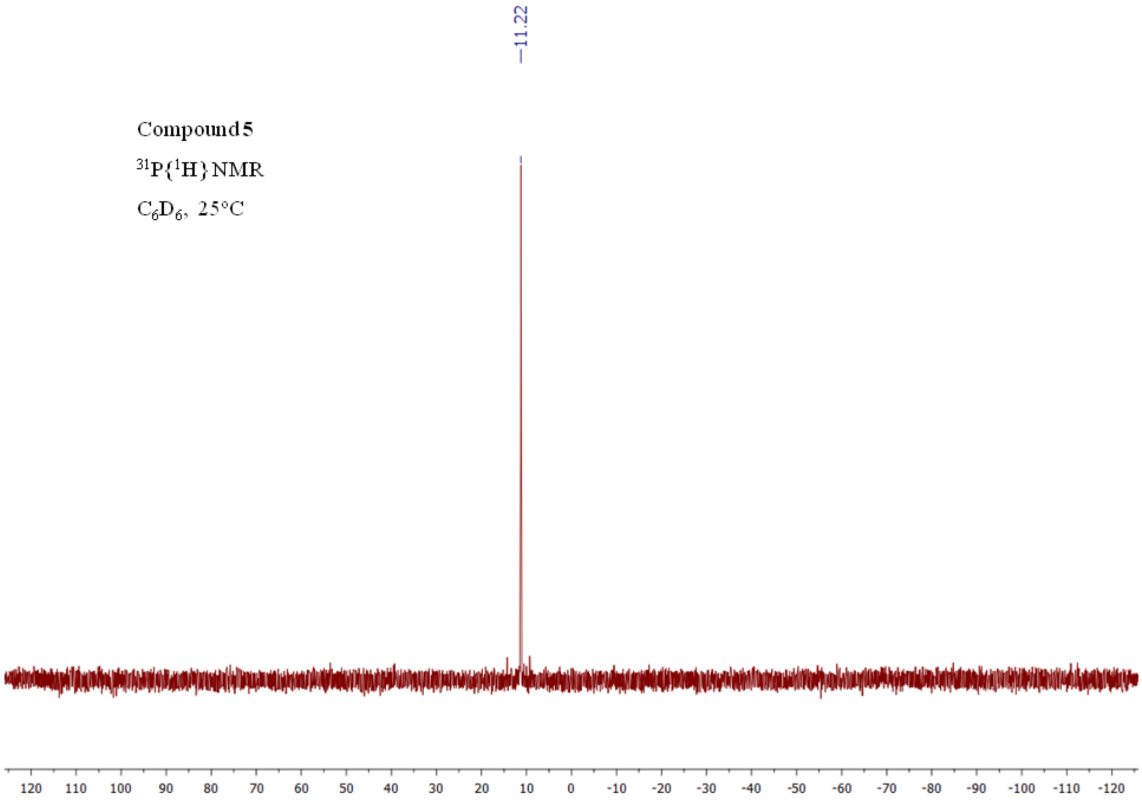
11.22
Compound 5
31P{1H} NMR
C6D6, 25°C
120 110 100 90 80 70 60 50 40 30 20 10 0 -10 -20 -30 -40 -50 -60 -70 -80 -90 -100 -110 -120

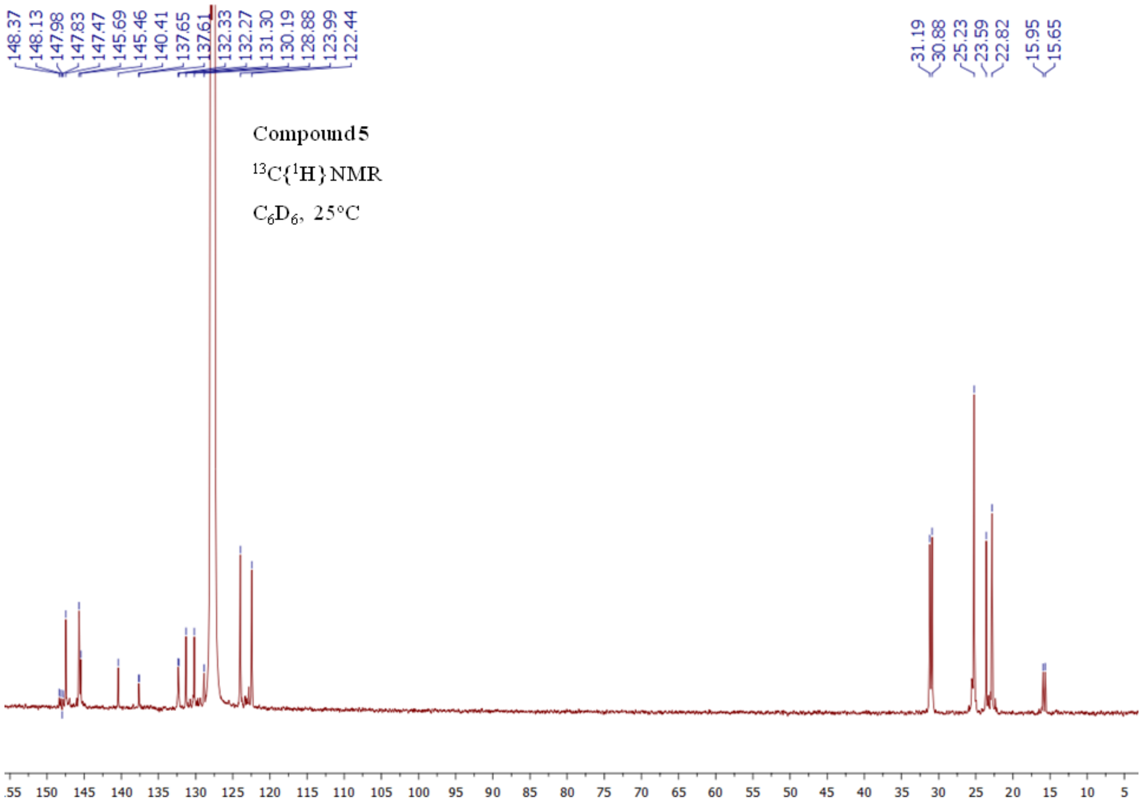
148.37
148.13
147.98
147.83
147.47
145.69
145.46
140.41
137.65
137.61
132.33
132.27
131.30
130.19
128.88
123.99
122.44
31.19
30.88
25.23
23.59
22.82
15.95
15.65
Compound 5
13C{1H} NMR
C6D6, 25°C

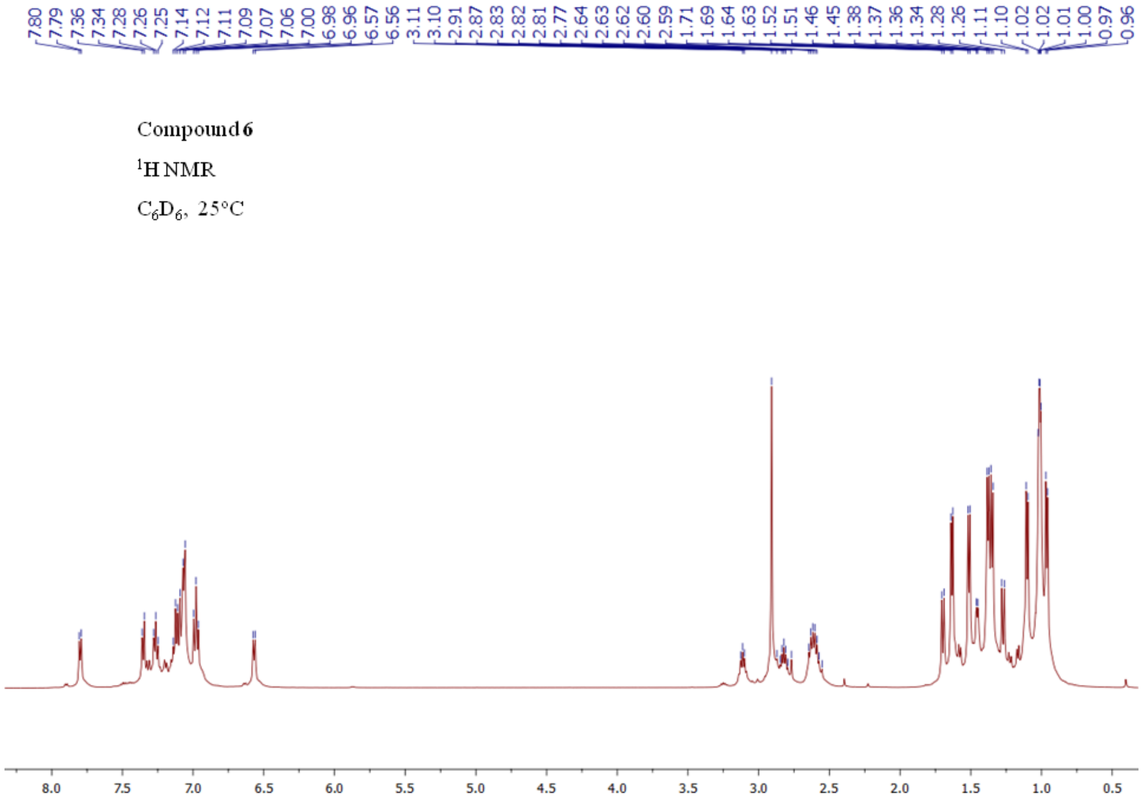
7.80
7.79
7.36
7.34
7.28
7.26
7.25
7.14
7.12
7.11
7.09
7.07
7.06
7.00
6.98
6.96
6.57
6.56
3.11
3.10
2.91
2.87
2.83
2.82
2.81
2.77
2.64
2.63
2.62
2.60
2.59
1.71
1.69
1.64
1.63
1.52
1.51
1.46
1.45
1.38
1.37
1.36
1.34
1.28
1.26
1.11
1.10
1.02
1.02
1.01
1.00
0.97
0.96
Compound 6
1H NMR
C6D6, 25°C

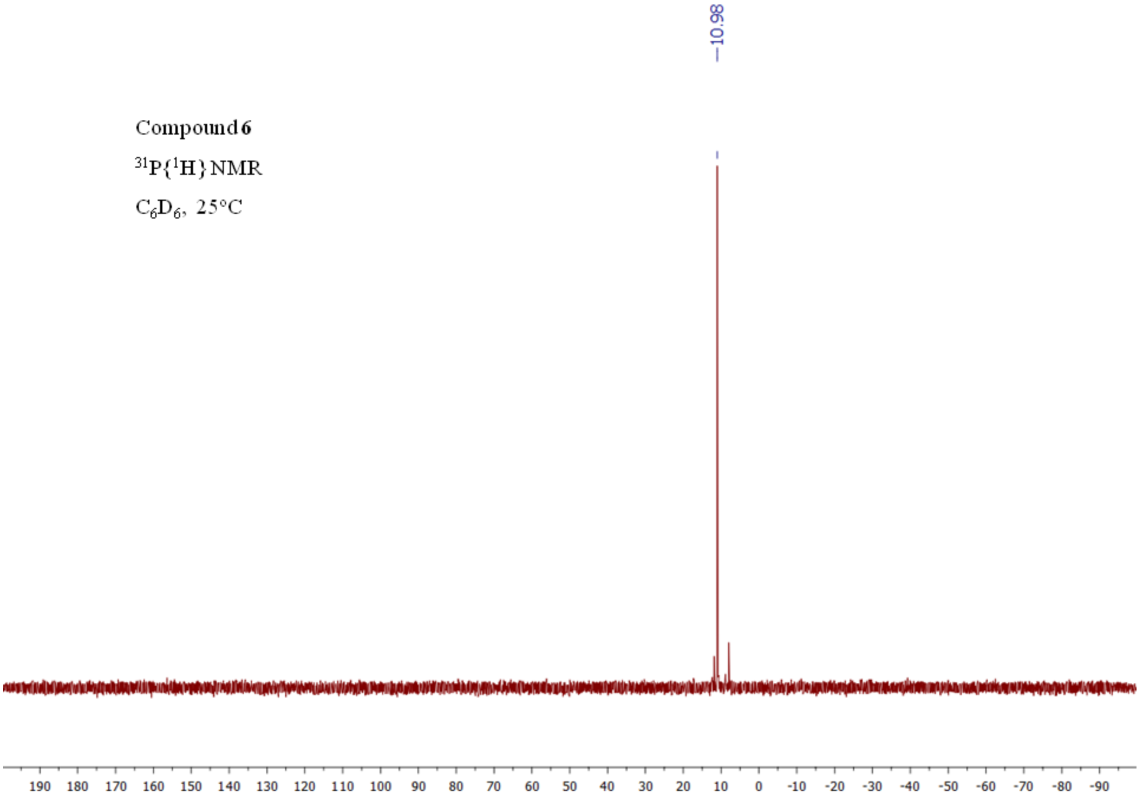
Compound 6
$^{31}P\{^1H\}$ NMR
C_6D_6, 25°C
10.98
190 180 170 160 150 140 130 120 110 100 90 80 70 60 50 40 30 20 10 0 -10 -20 -30 -40 -50 -60 -70 -80 -90

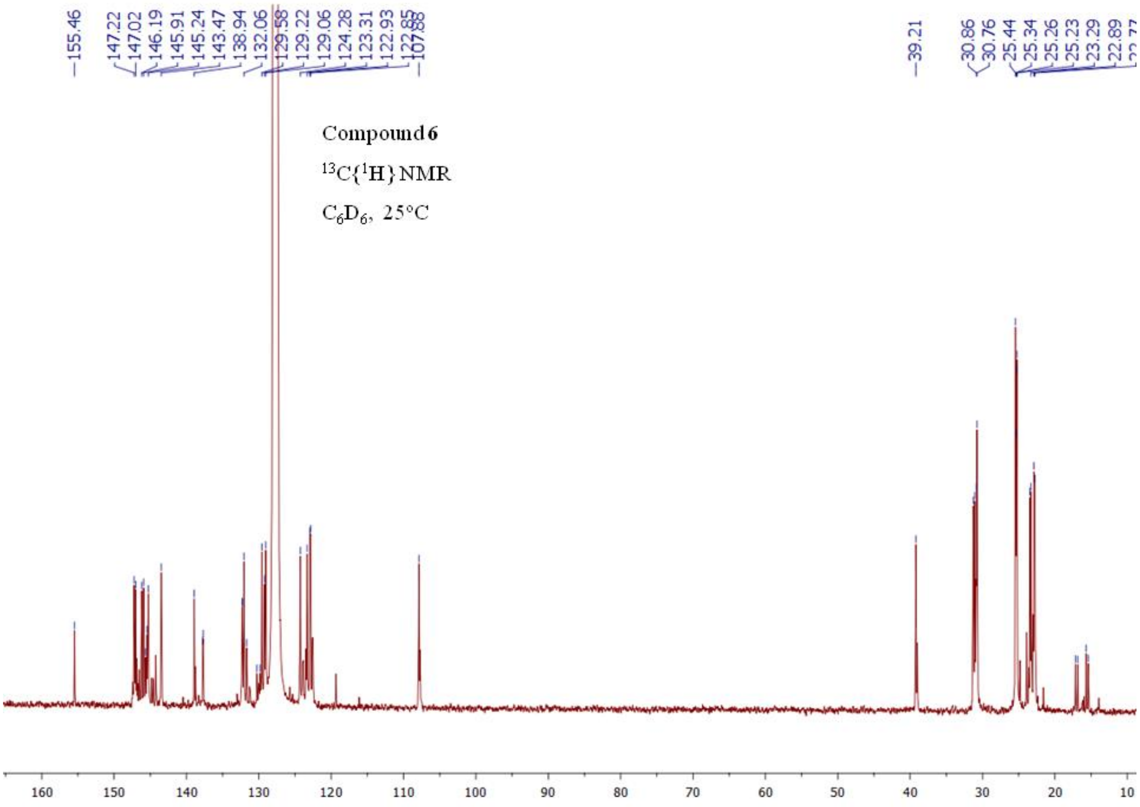
Compound 6
$^{13}C\{^1H\}$ NMR
C_6D_6, 25°C
155.46
147.22
147.02
146.19
145.91
145.24
143.47
138.94
132.06
129.58
129.22
129.06
124.28
123.31
122.93
39.21
30.86
30.76
25.44
25.34
25.26
25.23
23.29
22.89
160 150 140 130 120 110 100 90 80 70 60 50 40 30 20 10

6. References

[1] Pu, L.; Phillips, A. D.; Richards, A. F.; Stender, M.; Simons, R. S.; Olmstead, M. M.; Power, P. P. Germanium and Tin Analogues of Alkynes and Their Reduction Products. *J. Am. Chem. Soc.* **2003**, *125*, 11626-11636.

2 Espada, M. F.; Campos, J.; López-Serrano, J.; Poveda, M. L.; Carmona, E. Methyl-, Ethenyl-, and Ethynyl-Bridged Cationic Digold Complexes Stabilized by Coordination to a Bulky Terphenylphosphine Ligand. *Angew. Chem. Int. Ed.* **2015**, *54*, 15379-15384.

3 Martínez-Martínez, A. J.; Weller, A. S. Solvent-free anhydrous Li^+, Na^+ and K^+ salts of $[B(3,5\text{-}(CF_3)_2C_6H_3)_4]^-$, $[BArF4]^-$. Improved synthesis and solid-state structures. *Dalton Trans.* **2019**, ***48***, 3551-3554.

[4] See for example: (a) Tang, Y.; Yu, B. Coinage Metal (Bisfluorosulfonyl)imide Complexes: Preparation, Characterization, and Catalytic Applications. *Eur. J. Inorg. Chem.* **2020**, 107–118; (b) Nurdin, L.; Spasyuk, D. M.; Fairburn, L.; Piers, W. E.; Maron, L. Oxygen−Oxygen Bond Cleavage and Formation in Co(II)-Mediated Stoichiometric O_2 Reduction via the Potential Intermediacy of a Co(IV) Oxyl Radical. *J. Am. Chem. Soc.* **2018**, *140*, 16094−16105; (c) Bohle, D. S.; Chua, Z. Iridium(I) Complexes of π-Acidic Carboxamides. *Organometallics* **2015**, *34*, 1074−1084; (d) Bowring, M. A.; Bergman, R. G.; Tilley, T. D. Pt-Catalyzed C−C Activation Induced by C−H Activation. *J. Am. Chem. Soc.* **2013**, *135*, 13121−13128.

[5]Hidalgo, N.; Bajo, S.; Moreno, J. J.; Navarro-Gilabert, C.; Mercado, B.; Campos, J. Reactivity of a Gold(I)/Platinum(0) Frustrated Lewis Pair with Germanium and Tin Dihalides. *Dalton Trans.* **2019**, *48*, 9127-9138.

[6] See for example: (a) Honeyman, C. H.; Lough, A. J.; Manners, I. Synthesis and Structures of the Halogenated Tungsten(VI) Phosphoraniminate Complexes WC15(N=PC13) and WCl4(N=PCl2Ph)2 and the Weakly Coordinated Ion Pair [WCl4(N=PCl3)][GaCl4]. *Inorg. Chem.* **1994**, *33*, 2988-2993. (b) Borovik, A. S.; Bott, S. G.; Barron, A. R. Arene-Mercury Complexes Stabilized Aluminum and Gallium Chloride: Synthesis and Structural Characterization. *J. Am. Chem. Soc.* **2001**, *123*, 11219-11228; (c) Branch, C. S.; Barron, A. R. Arene-Mercury Complexes Stabilized by Gallium Chloride: Relative Rates of H/D and Arene Exchange. *J. Am. Chem. Soc.* **2002**, *124*, 14156-14161; (d) Forfar, L. C.; Clark, T. J.; Green, M.; Mansell, S. M.; Russell, C. A.; Sanguramatha, R. A.; Slattery, J. M. White phosphorus as a ligand for the coinage metals. *Chem. Commun.* **2012**, *48*, 1970–1972; (e) Joost, M.; Mallet-Ladeira, S.; Miqueu, K.; Amgoune, A.; Bourissou, D. σ-SiH Complexes of Copper: Experimental Evidence and Computational Analysis. *Organometallics* **2013**, *32*, 898−902; (f) Margulieux, K. R.; Sun, C.; Kihara, M. T.; Colson, A. C.; Zakharov, L. N.; Whitmire, K. H.; Holland, A. W.; Pak, J. J. Synthesis and Characterization of Bimetallic Single-Source Precursors $(Ph_3P)_2M(\mu\text{-}SEt)_2E(SEt)_2$ for MES_2 Chalcopyrite Materials (M = Cu, Ag and E = In, Ga, Al). *Eur. J. Inorg. Chem.* **2017**, 2068–2077.

[7] (a) Wang, G.; Ceylan, Y. S.; Cundari, T. R.; Dias, H. V. R. Heterobimetallic Silver−Iron Complexes Involving Fe(CO)5 Ligands. *J. Am. Chem. Soc.* **2017**, *139*, 14292−14301; (b) Smith, J. B.; Kerr, S. H.; White, P. S.; Miller, A. J. M. Thermodynamic Studies of Cation−Macrocycle Interactions in Nickel Pincer−Crown Ether Complexes Enable Switchable Ligation. *Organometallics* **2017**, *36*, 3094−3103; (c) Chadwick, F. M.; Rees, N. H.; Weller, A. S.; Krämer, T.; Iannuzzi, M.; Macgregor, S. A. A Rhodium–Pentane Sigma-Alkane Complex: Characterization in the Solid State by Experimental and Computational Techniques *Angew. Chem. Int. Ed.* **2016**, *55*, 3677 –3681; (d) Pike, S. D.; Chadwick, F. M.; Rees, N. H.; Scott, M. P.; Weller, A. S.; Krämer, T.; Macgregor, S. A. Solid-State Synthesis and Characterization of σ-Alkane Complexes, $[Rh(L_2)(\eta^2,\eta^2\text{-}C_7H_{12})][BAr_{F4}]$ (L_2 = Bidentate Chelating Phosphine). *J. Am. Chem. Soc.* **2015**, *137*, 820−833; (e) Pike, S. D.; Pernik, I.; Theron, R.; McIndoe, J. S.; Weller, A. S. Relative binding affinities of fluorobenzene ligands in cationic rhodium bisphosphine η^6-fluorobenzene complexes probed using collision-induced dissociation. *Journal of Organometallic Chemistry*, **2015**, *784*, 75 - 83; (f) Pike, S. D.; Everett, M.; Jolleys, A.; Levason, W.; Pugh, D.; Reid, G. Unexpected neutral aza-macrocycle complexes of sodium. *Chem. Commun.* **2014**, *50*, 5843 - 5846; (g) Thompson, A. L.; Algarra, A. G.; Apperley, D. C.; Macgregor, S. A.; Weller, A. S. Synthesis and Characterization of a Rhodium(l) a-Alkane Complex in the Solid State. *Science*, **2012**, *337*, 1648 - 1651; (h) Moret, S.; Dallanegra, R.; Chaplin, A. B.; Douglas, T. M.; Hiney, R. M.; Weller, A. S. Alkyl dehydrogenation in iridium tri-cyclopentyl phosphines. *Inorganica Chimica Acta* **2010**, *363*, 574–580; (i) O'Connor, A. R.; White, P. S.; Brookhart, M. Synthesis, Characterization, and Reactivity Studies of (Cyclohexenyl)nickel(II) Complexes. *Organometallics* **2010**, *29*, 5382–5389; (j) O'Connor, A. R.; Brookhart, M. Polymerization of 1,3-Dienes and Styrene Catalyzed by Cationic Allyl Ni(II) Complexes. *Polymer Chemistry* **2010**, *48*, 1901 – 1912; (k) Douglas, T. M.; Molinos, E.; Brayshaw, S. K.; Weller, A. S. Rhodium Phosphine Olefin Complexes of the Weakly Coordinating Anions $[BArF_4]^-$ and $[1\text{-}closo\text{-}CB_{11}H_6Br_6]^-$. Kinetic versus Thermodynamic Factors in Anion Coordination and Complex Reactivity. *Organometallics* **2007**, *26*, 463 - 465.

[8] Claridge, T. High Resolution NMR Techniques in Organic Chemistry; Elsevier 2008, Chapter 8

[9] (a) Hoffman, R. E.; Shabtai, E.; Rabinovitz, M.; Iyer, V. S.; Müllen, K.; Rai, A. K.; Bayrd, E.; Scott, L. T. Self-diffusion measurements of polycyclic aromatic hydrocarbon alkali metal salts *J. Chem. Soc., Perkin Trans.* **1998**, *2*, 1659-1664; (b) Cohen, Y.; Ayalon, A. Self-Diffusion of Charged Polycyclic Systems and Their Parent Compounds: A PGSE NMR Study. *Ang. Chem. Int. Ed.* **1995**, *34*, 816-818.

[10] (a) Duttwyler, S.; Do, Q.-Q.; Linden, A.; Baldridge, K. K.; Siegel, J. S. Synthesis of 2,6-Diarylphenyldimethylsilyl Cations: Polar-π Distribution of Cation Character. *Angew. Chem. Int. Ed.* **2008**, *47*, 1719 –1722; (b) Li, J.; Schenk, C.; Winter, F.; Scherer, H.; Trapp, N.; Higelin, A.; Keller, S.; Pottgen, R.; Krossing, I.; Jones, C. Weak Arene Stabilization of Bulky Amido-Germanium(II) and Tin(II) Monocations. *Angew. Chem.Int. Ed.* **2012**, *51*, 9557 –9561. (c) Gerdes, C.; Saak, W.; Haase, D.; Müller, T. Dibenzosilanorbornadienyl cations and their Fragmentation into Silyliumylidenes. *J. Am. Chem. Soc.* **2013**, *135*, 10353−10361. (d) Sindlinger, C. P.; Aicher, F. S. W.; Wesemann, L. Cationic Stannylenes: In Situ Generation and NMR Spectroscopic Characterization. *Inorg. Chem.* **2017**, *56*, 548−560.

[11] Sheldrick, G. M. Crystal structure refinement with SHELXL. *Acta Cryst.* **2008**, A64, 112–122.

[12] (a) Steiner, T. Unrolling the hydrogen bond properties of C–H···O interactions. *Chem. Commun.* **1997** 727-734; (b) Desiraju, G. R. The C-H-O Hydrogen Bond in Crystals: What Is It? *Acc. Chem. Res.* **1991**, *24*, 290-296; (c) Gu, Y.; Kar, T.; Scheiner, S. Fundamental Properties of the CH···O Interaction: Is It a True Hydrogen Bond? *J. Am. Chem. Soc.* **1999**, *121*, 9411-9422; (d) Levina, E. O.; Chernyshov, I. Y.; Voronin, A. P.; Alekseiko, L. N.; Stash, A. I.; Vener, M. V. Solving the enigma of weak fluorine contacts in the solid state: a periodic DFT study of fluorinated organic crystals. *RSC Adv.* **2019**, *9*, 12520; (e) Balamurugan, V.; Hundal, M. S.; Mukherjee, R. First Systematic Investigation of C-H···Cl Hydrogen Bonding Using Inorganic Supramolecular Synthons: Lamellar, Stitched Stair-Case, Linked-Ladder, and Helical Structures. *Chem. Eur. J.* **2004**, *10*, 1683-1690.

[13] Gaussian 09, Revisions E.01 and B.01, M. J. Frisch, G. W. Trucks, H. B. Schlegel, G. E. Scuseria, M. A. Robb, J. R. Cheeseman, G. Scalmani, V. Barone, B. Mennucci, G. A. Petersson, H. Nakatsuji, M. Caricato, X. Li, H. P. Hratchian, A. F. Izmaylov, J. Bloino, G. Zheng, J. L. Sonnenberg, M. Hada, M. Ehara, K. Toyota, R. Fukuda, J. Hasegawa, M. Ishida, T. Nakajima, Y. Honda, O. Kitao, H. Nakai, T. Vreven, J. A. Montgomery, Jr., J. E. Peralta, F. Ogliaro, M. Bearpark, J. J. Heyd, E. Brothers, K. N. Kudin, V. N. Staroverov, T. Keith, R. Kobayashi, J. Normand, K. Raghavachari, A. Rendell, J. C. Burant, S. S. Iyengar, J. Tomasi, M. Cossi, N. Rega, J. M. Millam, M. Klene, J. E. Knox, J. B. Cross, V. Bakken, C. Adamo, J. Jaramillo, R. Gomperts, R. E. Stratmann, O. Yazyev, A. J. Austin, R. Cammi, C. Pomelli, J. W. Ochterski, R. L. Martin, K. Morokuma, V. G. Zakrzewski, G. A. Voth, P. Salvador, J. J. Dannenberg, S. Dapprich, A. D. Daniels, O. Farkas, J. B. Foresman, J. V. Ortiz, J. Cioslowski, and D. J. Fox, Gaussian, Inc., Wallingford CT, 2010.

[14] Adamo, C.; Barone, V. Toward reliable density functional methods without adjustable parameters: The PBE0 model, *J. Chem. Phys.*, **1999**, *110*, 6158-69.

[15] Grimme, S.; Ehrlich, S.; Goerigk, L. Effect of the damping function in dispersion corrected density functional theory, *J. Comp. Chem*. **2011**, *32*, 1456-65.

[16] (a) Ditchfield, R.; Hehre, W. J.; Pople. J. A. Self-Consistent Molecular-Orbital Methods. IX. An Extended Gaussian-Type Basis for Molecular-Orbital Studies of Organic Molecules. *J. Chem. Phys.* **1971**, *54*, 724–728; (b) Hehre, W. J.; Ditchfield, R.; Pople J. A. Self—Consistent Molecular Orbital Methods. XII. Further Extensions of Gaussian-Type Basis Sets for Use in Molecular Orbital Studies of Organic Molecules. *J. Chem. Phys.* **1972**, *56*, 2257–2261; (c) Hariharan, P. C.; Pople, J. A. The Influence of Polarization Functions on Molecular Orbital Hydrogenation Energies. *Theor. Chim. Acta* **1973**, *28*, 213–222; (d) Francl, M. M.; Pietro, W. J.; Hehre, W. J.; Binkley, J. S.; Gordon, M. S.; DeFrees, D. J.; Pople. J. A. Self-Consistent Molecular Orbital Methods. XXIII. A Polarization-Type Basis Set for Second-Row Elements. *J. Chem. Phys.* **1982**, *77*, 3654–3665.

[17] Andrae, D.; Haeussermann, U.; Dolg, M.; Stoll, H.; Preuss, H. Energy-Adjusted Ab Initio Pseudopotentials for the Second and Third Row Transition Elements, *Theor. Chim. Acta* **1990**, *77*, 123–141.

[18] Marenich, A. V.; Cramer, C. J.; Truhlar, D. G. Universal Solvation Model Based on Solute Electron Density and on a Continuum Model of the Solvent Defined by the Bulk Dielectric Constant and Atomic Surface Tensions, *J. Phys. Chem. B* **2009**, *113*, 6378–6396.

[19] Weigend, F.; Ahlrichs, R. Balanced basis sets of split valence, triple zeta valence and quadruple zeta valence quality for H to Rn: Design and assessment of accuracy, *Phys. Chem. Chem. Phys.* **2005**, *7*, 329.

[20] Andrae, D., Häußermann, U., Dolg, M., Stoll, H., Preuß, H., Energy-adjusted initio pseudopotentials for the second and third row transition elements, *Theor. Chim. Acta,* **1990**, *77*, 123-141.

[21] (a) Multiwfn, v. 6.0. http://sobereva.com/multiwfn/; (b) Lu, T.; Chen, F. Multiwfn: A multifunctional wavefunction analyzer. *J. Comput. Chem.* **2012**, *33*, 580-592.

[22] (a) Glendening, E. D.; Landis, C. R.; Weinhold, F. NBO 6.0 : Natural bond orbital analysis program. *J. Comput. Chem.* **2013**, *34*, 1429-1437; (b) Glendening, E. D.; Badenhoop, J. K.; Reed, A. E.; Carpenter, J. E.; Bohmann, J. A.; Morales, C. M.; Landis, C. R.; Weinhold, F. NBO 6.0.; Theoretical Chemistry Institute, University of Wisconsin: Madi-son, **2013**. Available at: www.chem.wisc.edu.

[23] CYLview, 1.0b; Legault, C. Y., Université de Sherbrooke, 2009 (http://www.cylview.org).

[24] (a) Bader, R. F. W. Atoms in Molecules: A Quantum Theory, Oxford University Press, Oxford, **1995**; (b) Bader, R. F. W. A Quantum Theory of Molecular Structure and Its Applications, *Chem. Rev.* **1991**, *91*, 893-928.
